# Processability of Different Polymer Fractions Recovered from Mixed Wastes and Determination of Material Properties for Recycling

**DOI:** 10.3390/polym13030457

**Published:** 2021-01-31

**Authors:** Selina Möllnitz, Michael Feuchter, Ivica Duretek, Gerald Schmidt, Roland Pomberger, Renato Sarc

**Affiliations:** 1 Department of Environmental and Energy Process Engineering, Chair of Waste Processing Technology and Waste Management, Montanuniversitaet Leoben—Franz-Josef-Straße 18, 8700 Leoben, Austria; selina.moellnitz@unileoben.ac.at (S.M.); roland.pomberger@unileoben.ac.at (R.P.); 2 Department of Polymer Engineering, Chair of Materials Science and Testing of Polymers, Montanuniversitaet Leoben—Otto Glöckel-Straße 2, 8700 Leoben, Austria; michael.feuchter@unileoben.ac.at; 3 Department of Polymer Engineering, Chair of Polymer Processing, Montanuniversitaet Leoben—Otto Glöckel-Straße 2, 8700 Leoben, Austria; ivica.duretek@unileoben.ac.at; 4 Saubermacher Dienstleistungs AG, Hans-Roth-Straße 1, 8073 Feldkirchen bei Graz, Austria; G.Schmidt@saubermacher.at

**Keywords:** mixed wastes, polymer recycling, processability, material characterisation, material properties, circular economy

## Abstract

To achieve future recycling targets and CO_2_ and waste reduction, the transfer of plastic contained in mixed waste from thermal recovery to mechanical recycling is a promising option. This requires extensive knowledge of the necessary processing depth of mixed wastes to enrich plastics and their processability in polymer processing machines. Also, the selection of a suitable processing method and product application area requires appropriate material behaviour. This paper investigates these aspects for a commercial processed, mixed waste, and two different mixed polyolefin fractions. The wastes are processed at different depths (e.g., washed/not washed, sorted into polyethylene, polypropylene, polyethylene terephthalate, polystyrene/unsorted) and then either homogenised in the extruder in advance or processed heterogeneously in the compression moulding process into plates. The produced recyclates in plate form are then subjected to mechanical, thermal, and rheological characterisation. Most investigated materials could be processed with simple compression moulding. The results show that an upstream washing process improves the achievable material properties, but homogenisation does not necessarily lead to an improvement. It was also found that a higher treatment depth (recovery of plastic types) is not necessary. The investigations show that plastic waste recovery with simple treatment from mixed, contaminated wastes into at least downcycling products is possible.

## 1. Introduction

The waste management industry often talks about “plastics” as if it were a single material, but this is not the case. Plastics are an extensive family of entirely different materials. Each plastic type is designed with specific characteristics that make it ideal for its intended application. Whatever their application was, at the end of their service life, plastic materials are necessary resources that should first be recycled (upstream/downstream), and only when this is no longer technically possible and economically feasible, they should be used as an alternative energy source in energy recovery facilities [1,2,3].

In 2019, 368 million tonnes (Mt) of plastic were produced worldwide—57.9 Mt in Europe (EU28+NO/CH) [1], and the EU converters’ demand was about 50.7 Mt. “Packaging” (~40%) and “building and construction” (~20%) represent the largest end-use markets followed by the “automotive industry” with about 10% [4]. The most frequently used plastic types are the polyolefins (PO) (polyethylene—PE; polypropylene—PP) at approx. 50% [4]. These are mainly used in the packaging sector, e.g., food packaging, hinged caps, bags, trays, films, and bottles, but also for pipes, automotive parts, agricultural films, houseware parts, etc. Other common packaging plastics are polyethylene terephthalate (PET) at about 8% and polystyrene (PS) at about 6.5% [4]. The main areas of PET application are the production of fibres for the textile industry, moulding compounds, hollow bodies, and films, primarily for the packaging sector. Standard PS is mainly used to produce dimensionally stable food and other packaging products, e.g., cups, trays, caps, closures, boxes, and films. Expanded PS (EPS) and extruded PS (XPS) are also frequently used as insulation material for thermal insulation or impact sound insulation.

In 2018, 29.1 Mt plastic post-consumer waste was collected in the (EU28+NO/CH) [4], which ended up in three different waste management paths: 42.6% were used as SRF (solid recovered fuel) for energy recovery, 32.5% (81% within the EU) were recycled, and 24.9% were still landfilled in Europe [4]. However, the figures for the last ten years show that waste management is currently transforming in Central Europe, especially in Austria. It is further developing from a thermal recovery to a recycling economy [5]. Nowadays, most plastics present in mixed wastes like commercial and municipal solid waste end up as SRF in energy recovery and are irrevocably lost for recycling [6]. Only PET in bottle form for recycling and PVC (polyvinyl chloride) parts are discharged, representing a contaminant for further processing. According to the EU [7], municipal solid waste recycling rates of 65% are to be achieved by 2035. In 2018, 86.1% of mixed municipal solid waste (excluding bulky waste and separate collection) in Austria was treated thermally directly or after mechanical-biological waste treatment, and 12.3% was treated biologically. Only 1.6%, mainly metals and glass, were recycled [8]. The EU has released a plastic strategy that sets that by 2030, half of the plastic waste generated in the EU will be recycled. The sorting and recycling capacity has to be increased fourfold compared to the reference year 2015. Among others, future recycling rates can be met by upgrading relevant plastics from “Other recovery”, e.g., “energy recovery”, to “recycling” [3]. Recycled plastics are generally considered to be of lower quality than virgin plastics [1,2]. However, several key challenges need to be overcome. For a high recycled material quality, high purity of the input material is necessary, and external (e.g., glue) as well as internal impurities (e.g., adsorbed substances) must be removable. For pure, clean plastics, modern recycling processes can match virgin properties.

Nevertheless, many mixed waste streams (e.g., mixed commercial or municipal waste) are considered low value [9] and, therefore, not (economically) recyclable because of the high treatment costs or the high level of contamination [10]. Nevertheless, this does not mean that technical recycling is excluded. To investigate this, it is first of all necessary to determine whether a sufficient amount of plastic is contained in the mixed waste and whether separation is possible. If plastic mixtures or even individual types can be sorted out, the next step is to examine the processability with simple processing methods. If this is possible, a basic characterisation of the resulting materials follows. A suitable processing method can be selected only then, and the producible products can be determined [10].

The novelty of this research is the investigation of mixed, heterogenous, and contaminated (e.g., organic and inorganic impurities) mixtures with significant plastic amounts. Nowadays, such mixtures are declared as sorting residues (i.e., a non-recyclable fraction from material recovery facilities) and are utilised in energy recovery processes [10].

The plastic amount in the mixtures was investigated in two ways: on one side, at the polymer type (PE, PP, PET, and PS) level, and on the other side, as a varying mixture of unsorted polymers. Therefore, simple recoverability (i.e., sorting out with/without washing) and processability (i.e., compression moulding with/without homogenisation for production of recyclates) of the mentioned two ways were extensively investigated.

Next, the material properties of the plates produced from the recyclates were determined to create a material database for further research work in the linking of waste management with the plastic recycling sector. Finally, the applied strategy and the characterisation included have been widely studied and validated both at the industrial level and the research stage.

## 2. Materials and Methods

### 2.1. Materials and Sampling

A common mixed waste, i.e., SRF (approx. 200 kg) produced from pre-treated and untreated mixed wastes (mixed municipal waste, commercial waste, etc., excluding separately collected wastes such as lightweight packaging waste) from a production facility near the city Graz in Austria, was used for the studies. In spring 2018, the sample was taken from the falling material stream according to ÖNORM S 2123-3 [11]. The material sample was taken from the SRF processing line after pre-shredding (<500 mm), magnetic separation, and PVC separation using a NIR (near-infrared) sorter and had a particle size >100 mm.

At the same time, about 20 kg of a sample of a PO-rich waste fraction (sample name: PO_A) was taken at the same plant. According to ÖNORM S 2123-3 [11], the sample was taken from the falling material stream. The sampling is carried out after pre-shredding, magnetic separation, PVC discharge, separation of heavy materials by a wind sifter, and subsequent post-shredding (<35 mm).

A further PO sample (approx. 8 kg) (sample name: PO_B) was taken out from the wet-mechanical processing unit using a centrifugal force separator [12] with a particle size <30 mm. The input material was a mixture of common SRF, mixed plastics from light-weight packaging treatment, and mixed plastics from the industry.

**Figure A2:**
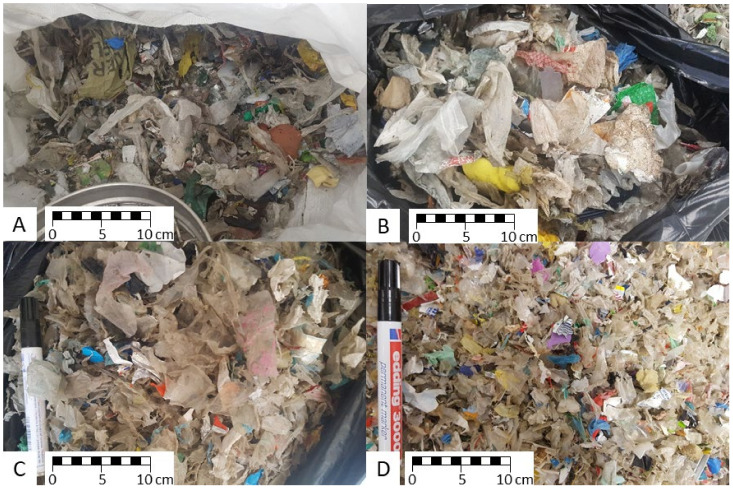
Exemplary photos of the input materials: SRF in (**A**) and (**B**), PO_A in (**C**), and PO_B in (**D**).

All samples mentioned are representative samples composed of individual increments taken continuously over several hours during the plant operation or test run. Exemplary photos of the three test materials are shown in Figure A2 in Appendix I.

### 2.2. Methods

**Figure 1:**
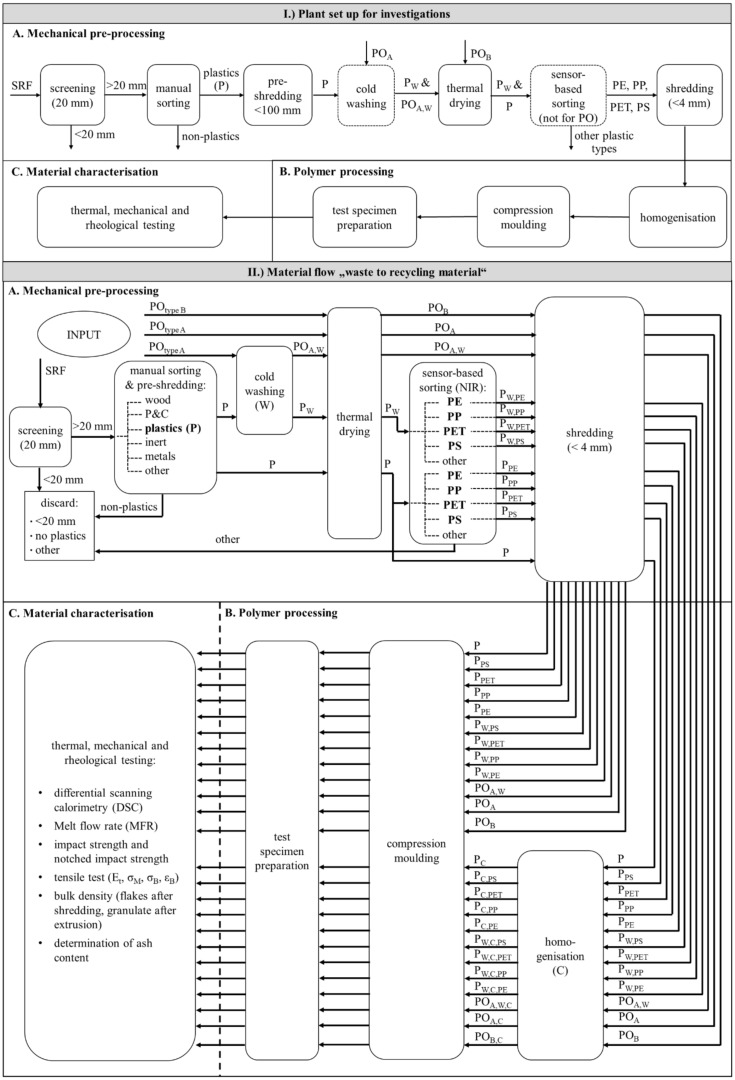
Flow chart for the plant set up of the investigations (**I**) and all material flows of input materials and resulting flows during the investigations (**II**); The process is divided into three sub-processes: **A**. Mechanical pre-processing, **B**. Polymer processing and **C**. Material characterisation.

In this section, the experimental and analytical procedures are described. Figure 1 (I. Plant set up for investigations) shows the modular plant configuration for material preparation, as it could also look like in real processing plants. The investigation method is divided into three areas: A.) Mechanical pre-processing of the input materials; B.) Polymer processing consisting of material homogenisation, a compression moulding process, and test specimen preparation; and C.) Material characterisation with thermal, mechanical, and rheological material testing.

The mechanical pre-processing consists of a drum screen for the separation of fine material (<20 mm), a manual sorting station for the removal of non-plastics and other materials, a double shaft pre-shredder to reduce the average particle size of plastics below 100 mm, a cold washing aggregate (a self-built stirred washer), a thermal drying cabinet (drying at 105 °C up to constant weight according to ONR CEN/TS 15414-1), a sensor-based sorting system (near-infrared) for the manual sorting of the standard plastic types (PE, PP, PET, and PS), and a post-shredder (cutting mill) to reduce the particle size to <4 mm.

In the polymer processing step, one-half of the shredded plastic flakes per plastic type were fed to a counter-rotating parallel twin-screw extruder TSE 42/7D (screw diameter (D): 42 mm; screw length: 7D; model no.: 8324; type: Plasti-Corder PL2000 from Brabender^®^ GmbH & Co. KG, Duisburg, Germany) with a three-zone screw for thermoplastics. This equipment is used for material homogenisation, e.g., thermoplastic multicomponent systems, polymer blends, or composite materials. The product (filament) was cooled in a water bath and granulated afterwards. The other half of the materials were directly processed into plates (dimensions: 160 mm × 160 mm × 4 mm) with a hot vacuum compression moulding process (vacuum press type P200PV, Dr. Collin GmbH, Maitenbeth, Germany). The material-specific four-zone temperature profile for material homogenisation is given in Table A3 in Appendix C. All materials were homogenised at a screw speed of 110 rpm. The material-specific, five-stage press profiles (temperature, pressure, and time) were determined empirically. The press profiles are presented in Table A4 in Appendix C.

Test specimens used for material characterisation were stamped or cut (CNC milling machine) from the plates. Extensive tests were carried out for this purpose: thermal characterisation with differential scanning calorimetry (DSC), melt mass flow rate (MFR), determination of ash content, mechanical characterisation with tensile tests, (notched) impact strength, and determination of the bulk density from plastic flakes after shredding and granulates after homogenisation.

Crystallinity ($X_{C}$) is calculated from the melting enthalpy ($\Delta H_{m}$) measured with DSC and the approximated melting enthalpy of totally crystalline material ($\Delta H_{0}$) from the literature according to Equation (1) [13,14].

(1) $$X_{C}=\frac{\Delta H_{m}}{\Delta H_{0}}\times 100\%$$

Figure 1 (II. Material flow “waste to recycling material”) shows all material flows generated during the investigations from waste to finished test specimens.

**Figure A3:**
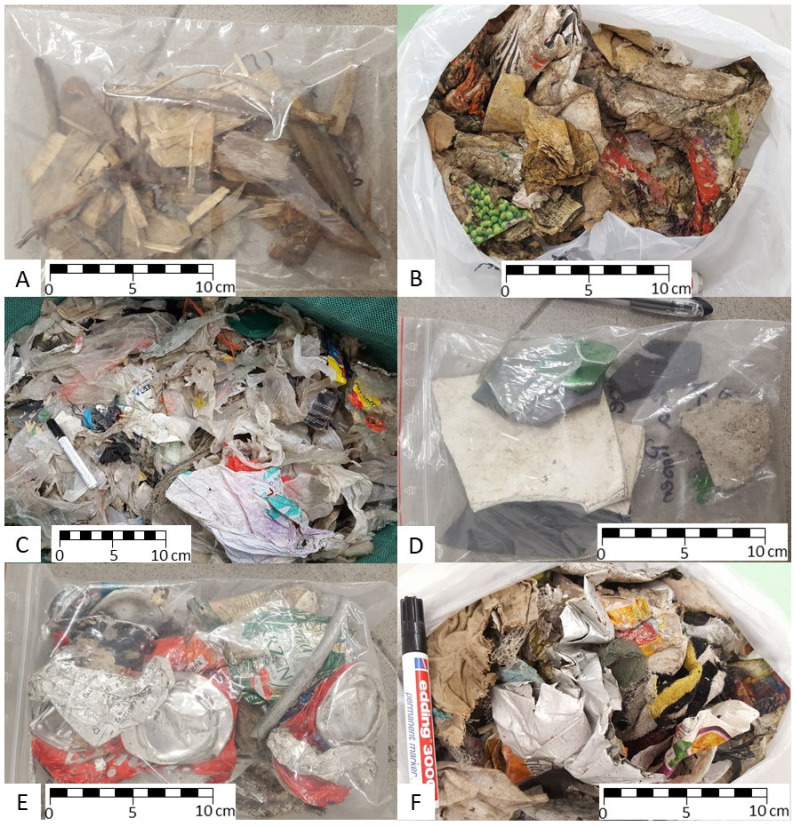
Exemplary photos of the manually sorted material fractions: wood—**A**; P&C—**B**; plastics—**C**; inert—**D**; metals—**E**; others—**F**.

**Figure A4:**
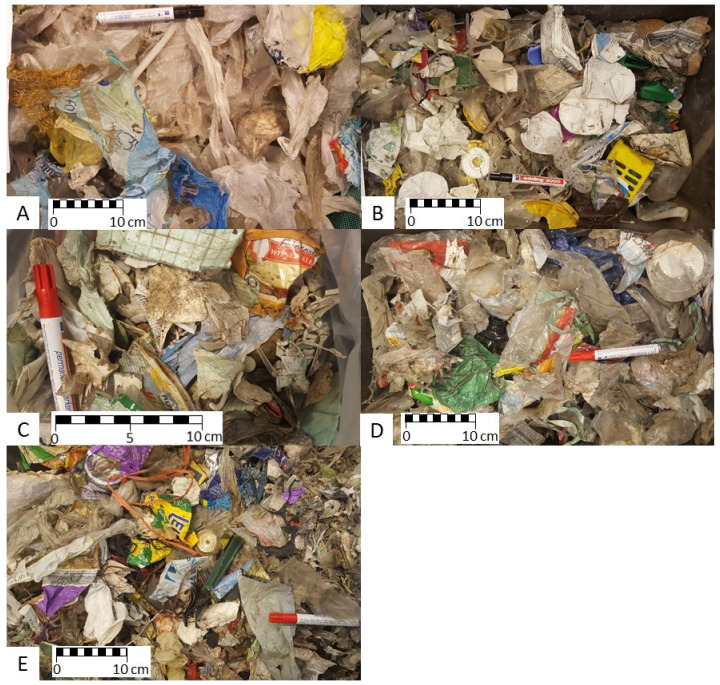
Exemplary photos of plastic types sorted with NIR: PE—**A**; PP—**B**; PET—**C**; PS—**D**; and others—**E**.

The mixed waste, i.e., SRF, was the only input material screened and manually sorted into six material fractions (wood, paper, and cardboard (P&C), plastics, inert, metals, and other materials). Exemplary photos of the manually sorted material fractions are shown in Appendix I in Figure A3. Only the plastic fraction was further processed and investigated. The other fractions were discarded and, therefore, not relevant for further investigation. The plastic fraction was divided into three similar parts. One part was dried and shredded without NIR sorting. The second part was dried and sorted by NIR into five plastic types and the rest. Exemplary photos of plastic types sorted with NIR are given in Appendix I in Figure A4. The third SRF part was washed, dried, and NIR-sorted. The sorted plastic types were post-shredded separately.

The two PO materials were not screened and not manually sorted due to their small grain size (i.e., <35 mm and <30 mm).

The input material PO_A was divided into two similar parts. One part was washed, dried, and shredded. The other part was dried and shredded without a washing step. As the input material, PO_B came from wet-mechanical processing, it was only dried and shredded.

All material flows were divided after shredding. Half of each material went into the extruder for homogenisation, and the other half was compression moulded directly into test plates without homogenisation.

#### **C.) Material Characterisation**

For thermal characterisation of the materials, DSC measurements were performed using a DSC1 (Mettler-Toledo GmbH, Urdorf, Switzerland) in a temperature range from 0 to 230 °C for PE, PP, and PS materials, and from 0 to 200 °C for PO and P materials with a heating rate of 10 K/min in a nitrogen atmosphere (nitrogen flux rate 50 mL/min). The cooling rate was 20 K/min. To make the thermal history the same for all materials, a measuring program with one heating, one cooling, and second heating was chosen. Only the cooling and second heating curves were used for analysis. In advance, for checking the thermal stability, measurements up to 300 °C with a heating rate of 20 K/min in a nitrogen atmosphere were carried out for each material. This was used to determine the range of measurement itself. Seven reproducibility measurements for the heterogeneous and three for the homogeneous materials were carried out according to DIN EN ISO 11357-1 [15]. Standard 40 µL aluminum crucibles with pierced lids were used.

Charpy impact tests and notched impact tests (Ceast Resil 25, INSTRON/Ceast, Pianezza, Italy) according to DIN EN ISO179-1 [16] were performed at room temperature using a pendulum with 2 J (unnotched) and 0.5 J (notched) for P_PE, P_W,PE, P_W,C,PE, P_W,PP, P_W,C,PP, PO_A,W, and PO_B,C; a pendulum with 0.5 J (notched and unnotched) for P_PP, P_C,PP, all PS materials, PO_A, PO_A,C, PO_A,W,C, PO_B, and P; and a 7.5 J pendulum (unnotched) for P_W,C,PE. Tensile tests (Zwick Z010, Zwick/Roell GmbH & Co. KG, Ulm, Germany) were performed at room temperature according to DIN EN ISO 527-1 [17] and EN ISO 527-2 [18]. The ash content was determined according to DIN EN ISO 3451-1 [19]. Due to the heterogeneity, three reproducibility measurements were carried out, and the mean values were calculated for the discussion. The bulk density was determined for the plastic flakes after shredding and for the granulates after compounding, respectively, according to DIN EN ISO 60 [20]. Five measurements per material were carried out. The MFR (Modular Melt (Mass) Flow Tester, INTERON/Ceast, I) was determined according to DIN EN ISO 1133-1 [21]. The test conditions were set to 190 °C and 2.16 kg for all materials except for the PS materials. For PS, the test conditions were set to 200 °C and 5 kg.

## 3. Results

For the examined plastic materials, the following properties were investigated: The composition of mixed waste, i.e., SRF and its plastic type content, thermal and mechanical properties, characterisation of the flow behaviour (MFR), bulk density, and ash content.

All stated values are wt.%_DS_ (DS—dry substance), given in full percent only for clarity purposes.

All PE, PP, PO, and P materials could be processed without any major problems. The PS materials emitted much gas in both processing variants, and several test runs were necessary to find a stable processing method. The PET materials could neither be homogenised nor compression moulded due to excessive contamination. Possible impurities are multilayer bottles, residual label material (PO), different non-compatible PET grades [10], diffused substances, etc. The reasons for non-processability were not further investigated in this paper. All other materials could be processed. The plates made out of the heterogeneous materials showed flow directions (see Appendix I: Figure A7, Figure A8 and Figure A9). These are due to material accumulations in the compression moulding process.

### 3.1. Total Composition of the Mixed Wastes and Plastic Type Content

The composition of the input materials does not influence the subsequent investigations and is given here only to complete the information. Further extensive and current data on typical SRF composition are given by [22]. The detailed data of the investigated material are given in Appendix A.

Of the fine material (<20 mm), 8.5%_OS_ (OS—original substance) was separated by pre-screening and discarded from SRF. The subsequent manual sorting analysis revealed the following composition of SRF > 20 mm. The plastics represented the largest material fraction with 86.5%_OS_. The other fraction (sorting residue and composites) represented 6.5%_OS_. The share of P&C was 5.8%_OS_. The share of metals and inert materials was 0.6%_OS_ each, and 0.1%_OS_ was the content of wood. The mass losses caused by material drying during storage, sorting losses (mobile organic material, dust formation, etc.), and screening losses were not taken into consideration for calculation here. These are in the range of 3%_OS_ of the total sample.

The sorted out plastic fraction (86.5%_OS_, see above) consisted of the following plastic types. The PE fraction represented the largest share with 36.4%_OS_. The other fraction (black and other plastics as well as unidentified objects) represented 21.2%_OS_. The PET share was 20.7%_OS,_ and PP was contained with 15.7%_OS_. The smallest fraction was PS with 6%_OS_.

### 3.2. Thermal Material Properties

Table 1 displays the evaluations of the DSC measurements. Evaluated were the crystallisation temperature (T_C_) with respective crystallisation enthalpy (ΔH_c_), melting temperatures (T_m1_ and T_m2_) with respective melting enthalpy (ΔH_m1_ and ΔH_m2_), and glass transition temperature (T_g_). A representative cooling curve and the second heating curve per analysed material for the respective material group (PE, PP, PS, PO, and P) are shown in Appendix B for better illustration.

The measured T_C_ for the investigated PE materials is, on average, 108 °C. The calculation of the crystallinity for the PE materials according to [13,14] with 293 J/g for totally crystalline PE resulted in values between 36% and 38% for a cooling rate of 20 K/min. This is a comparatively low crystallinity for PE and corresponds to that for virgin (v)LLDPE (10–50%) [23]. vLDPE typically has a crystallinity in the range of 45–55% and vHDPE in the range of 70–80% [23].

Two melting temperatures were determined for the PE materials. The primary melting point (T_m1_) is that most of the material melts are between 125 and 129 °C. The measured secondary melting point T_m2_ is about 162 °C and is due to contained impurities (higher melting foreign plastics such as PP, for example). Only P_W,PE shows a more distinct secondary melting point at approx. 110 °C. In the literature, melting temperature ranges for different vPE types are given as follows: 120–130 °C for LLDPE, 105–115 °C for LDPE, and 128–136 °C for HDPE [24]. It is interesting to note that the DSC curves are very similar, especially for the heterogeneous PE materials. As was to be expected, these become even more similar through homogenisation, which is evident in the smaller fluctuation margins.

The measured T_C_ for the investigated PP materials is 117 °C on average. According to [13,14], the calculation of the crystallinity with 207 J/g for totally crystalline PP yields values between 55% and 58% for a cooling rate of 20 K/min. This is a relatively high crystallinity for PP. Isotactic vPP has a crystallinity of 70–80%, syndiotactic PP of 30–40%, and atactic PP is amorphous and has no crystallinity [23,25]. The T_m1_ at approx. 165 °C was determined for the PP materials. Only P_PP and P_C,PP show a distinct T_m2_ at about 128 °C, which is due to contamination with foreign material, which can be removed by washing. In the literature, melting temperature ranges for vPP types are given between 161 and 186 °C [26]. Likewise, the DSC curves of the heterogeneous PP materials are very similar and, after homogenisation, even closer to each other.

Both heating curves of all investigated PS materials show a continuous decrease over the measured temperature range. This corresponds to the literature, as PS has low heat resistance, and from 55 °C onwards, an acceleration of ageing starts, which is why PS is usually only used up to 70 °C [27]. The measured T_g_ is about 99 °C on average, which corresponds to the literature value of about 80–100 °C for vPS [24,25]. The vPS types predominantly used are atactic and are, thus, in amorphous form and, therefore, have neither a T_C_ nor a T_m_ [24]. Therefore, it is remarkable that both a T_C_ (113–120 °C) and a T_m1_ (161–164 °C) were measured for the PS materials. The melting temperature is 240°C [28] for isotactic vPS and 270 °C [28] for syndiotactic vPS. The heterogeneous PS materials’ curves are much more heterogeneous compared to those of PE and PP and show more fluctuations and deviations from each other. Due to the homogenisation, these are smoothed considerably and are more similar to each other.

For the PO materials, a T_C_ at approx. 108 °C, a distinct T_m1_ at approx. 125 °C (ΔH_m_ = 50 J/g), and a T_m2_ at approx. 163 °C (ΔH_m_ = 15 J/g) are measured. Furthermore, a further secondary melting temperature is measured at approx. 110 °C. This has already been observed with P_W,PE. With PO_B,C, it is evident compared to the other PO materials that T_m1_ is more distinct and the secondary melting temperature at 110 °C is hardly present. Additionally, with PO_B,C, a second crystallisation peak at approx. 120 °C becomes clear from the HDPE content [24]. The comparison of the curves of the PO_A materials shows major deviations only for the cooling curves. The other curves are very similar, especially those of the homogeneous PO_A materials. For PO_B, the 2nd heating curves also show major deviations from each other.

The **mixed plastic fraction** (**P**) curves are surprisingly similar and show a high degree of similarity with those of PO materials. This is especially true for P_C and PO_B,C.

### 3.3. Melt Mass Flow Rate

**Figure 2:**
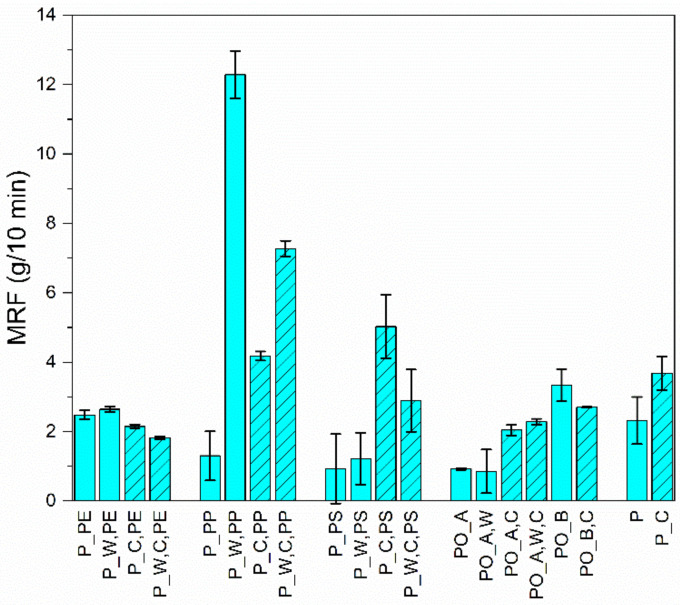
Averaged mass flow rate (MFR) values with standard deviation of all materials investigated.

Figure 2 shows a comparison of the mean MFR values of all materials investigated. The MFR results of all investigated materials are given in Appendix D.

### 3.4. Mechanical Material Properties

The measured MFR for the PE materials is between 1.8 and 2.6 g/10min. These are very low values. In the literature, MFR values between 0.5 and 25 g/10 min (test conditions: 190/2.16) are given for vLDPE and 0.35–17 g/10 min for vHDPE [24]. These are surprisingly good values, which indicate low material damage and, thus, good processability. No influence of the washing process can be seen.

PP materials show a significant increase in MFR due to washing. Thus, the MFR of P_PP is increased by a factor of 12 for P_W,PP. The MFR for P_C,PP is higher by a factor of three than for P_PP. The homogenised PP materials also show that a 40% higher MFR is achieved by washing. During all PP sample measurements, outgassing of volatile components was observed, which pushed the sample upwards [29,30]. This leads to certain measurement uncertainties. The reasons for this have not been further investigated in this paper. In the literature, MFR values of 0.5–65 (test conditions: 190/5) are found for vPP [24].

The large fluctuation ranges of all PS materials can be explained by the measurement uncertainties caused by clogging of the nozzle after a certain time. This was observed in all PS samples and can be explained by contaminants with a higher melting temperature [29]. The two heterogeneous PS materials show low MFR values compared to the homogenised PS materials. P_W,C,PS shows a lower MFR than P_C,PS. In the literature, MFR values between 1.5 and 18 g/10 min (test conditions: 200/5) are achieved for vPS [24]. Thus, the heterogeneous PS materials can be classified as very easy flowing and the homogeneous materials as normal flowing.

The heterogeneous PO_A materials show the lowest MFR values (below 1 g/10 min). There is no influence of washing on the MFR of the PO_A materials seen. Due to the homogenisation, the MFR rises to the MFR level of the PE materials. PO_B has a mean MFR of 3.3, which is reduced to 2.7 by homogenisation.

P has a mean MFR of 2.3, which is increased to 3.7 by homogenisation. The fluctuation ranges of the mean values can be explained by the measurement uncertainty caused by the outgassing of volatile components after a certain time [29,30]. The reasons for this have not been further investigated in this paper.

**Figure 3:**
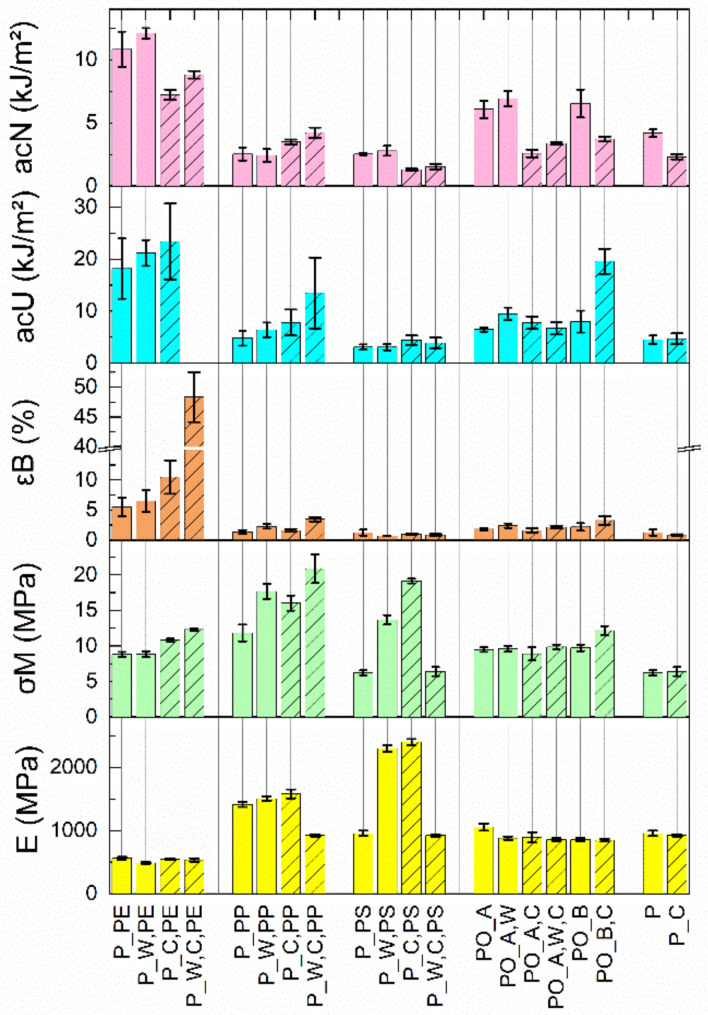
The results of the tensile tests (E, σ_M,_ ε_B_), the Charpy impact strength (a_cU_), and the Charpy notched impact strength (a_cN_) for all materials investigated.

The tensile parameters, the Charpy impact strength (a_cU_), and the Charpy notched impact strength (a_cN_) are reported in Figure 3 for all materials. The results of the impact tests of all investigated materials are given in Appendix E, and the results of the tensile tests are given in Appendix F.

Only P_W,C,PE displays plastic deformation with a well-defined yield point in the stress–strain curves [31]. P_PS, P_W,C,PP, P_PE, PO_A, and PO_A,C showed this behaviour only with single test specimens. The other materials showed mainly brittle behaviour [31].

The Young’s modulus (E) is for all PE materials in a similar range around 530 ± 40 MPa, and no significant influences due to washing or homogenisation can be detected. Comparison with data from the literature (vLDPE: ~200 MPa; vHDPE: ~1000 MPa) [24,31] shows that the achievable values are acceptable. According to the literature, vPE has the following tensile strengths (σ_M_) and elongations at the yield point (ε_M_): vLDPE—8–15 MPa at ~20%; and vHDPE—20–30 MPa at ~12% [24,31]. The literature gives elongations at break (ε_B_) of 400–800% [24]. The measured values for σ_M_ correspond to those for vLDPE. However, both ε_M_ and ε_B_ are far below the literature values. The notched impact strength for all PE materials is in the range between 6 and 15 kJ/m^2^. In the literature, values of about 6 kN/m^2^ or without a break are given for vPE [24,31]. For the impact strength, the literature predominantly states “no break”. The examined PE materials are mostly only partially broken, and P_W,C,PE is not broken at all (see notes in Table A6, Table A7, Table A8, Table A9, Table A10, Table A11, Table A12, Table A13, Table A14, Table A15, Table A16, Table A17, Table A18, Table A19, Table A20, Table A21, Table A22, Table A23, Table A24 and Table A25 in Appendix E) [32].

The PP materials have Young’s moduli (~1500 MPa) almost three times higher than PE. Interestingly, P_W,C,PP is the lowest value of this material series at 923 ± 19 MPa. Again, a comparison with the literature values (E: 1300–1800 MPa) [23,33] shows that these values are acceptable. For vPP, σ_M_ between 25 and 40 MPa at ε_M,_ around 20% [24], depending on the type, can be found in the literature. Depending on the vPP type, ε_B_ of 200–900% is possible [24]. The measured σ_M_ for PP materials is below 25 MPa, and only an ε_B_ of 2.5 ± 0.4% was measured. As with the PE materials, this indicates significant material embrittlement. The impact strength determined for the PP materials is below 20 kJ/m^2^, which corresponds to the literature values [24]. The a_cN_ determined is between 2 and 5 kJ/m^2^, which is slightly below the literature’s values (vPP: 4–12 kJ/m^2^) [24].

The Young’s modulus of P_PS (~955 MPa) shows a sharp increase to ~2300 MPa for P_W,PS and about 2400 MPa for P_C,PS and thus, are the highest values of all materials examined. However, the combination of washing and homogenisation causes E (~923 MPa) to drop even below the initial value of P_PS. In the literature, values between 2200 and 3300 MPa [24] are given for vPS. For vPS, σ_B_ between 45 and 65 MPa/mm^2^ and 3 and 4% for ε_B_ are found in the literature [24,33]. The measured a_cU_ of the PS materials is below 5 kJ/m^2^ and below the values found in the literature (5–20 kJ/mm^2^) for vPS [31,33]. The a_cN_ of the PS materials are between 1 and 2.5 kJ/m^2^, and this is in the field of the literature values (vPS: ~2.0 kJ/m^2^) [24].

Except for PO_A (1,053 ± 56 MPa), the PO materials have very similar Young’s moduli between 830 and 900 MPa. Significant influences due to washing or homogenisation are not recognisable. The Young’s moduli of the P and P_C material (~940 MPa) are also very similar, and no influence of homogenisation can be seen.

The values of σ_M_ and ε_B_ increases due to the homogenisation of PE, PP, and PO_B materials. With PS, a significant increase is measured of σ_M_ and ε_M_ by washing or compounding, but in combination, no significant change to P_PS is observed. The PO_A materials all show very similar values for σ_M_ and ε_M,_ with the higher values for PO_A,W,C being achieved.

### 3.5. Ash Content

**Figure 4:**
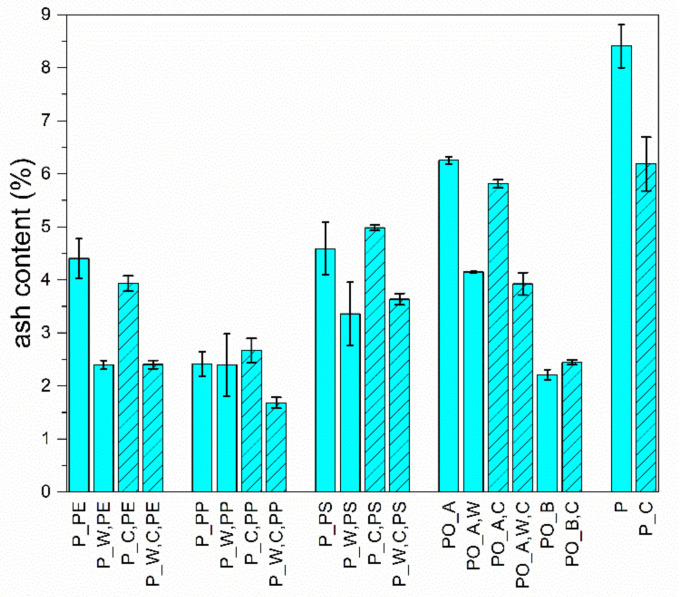
Calculated ash contents of all investigated materials.

Figure 4 shows the ash contents (AC) of all investigated materials. The results of the ash content measurements of all investigated materials are given in Appendix H.

The AC for the PE materials decreases for both the heterogeneous and the homogeneous fraction from about 4% to 2.4% by about 40% due to the washing process. For the two heterogeneous PP materials, no influence of the washing on the AC of about 2.4% was observed. In the homogenised PP fraction, the AC decreases by approx. 40% from 2.7% to 1.7% due to washing. The AC of the PS and PO_A materials has been reduced by approx. 30% for both the heterogeneous and the homogeneous fraction by washing. The unwashed PO_A materials have the second-highest AC of all investigated materials. The average AC for the PO_B is 2.2%, and for PO_B,C 2.5%. The lower contents compared to PO_A can be explained by the cleaner input materials (e.g., pre-sorted mixed plastic fraction) used for PO_B production. As expected, the unwashed, unsorted mixed plastic fraction (P) has the highest AC (approx. 8.4% for P and 6.2% for P_C) since there was no surface cleaning by washing or losing fine material, e.g., by sorting.

### 3.6. Bulk Densities

**Figure 5:**
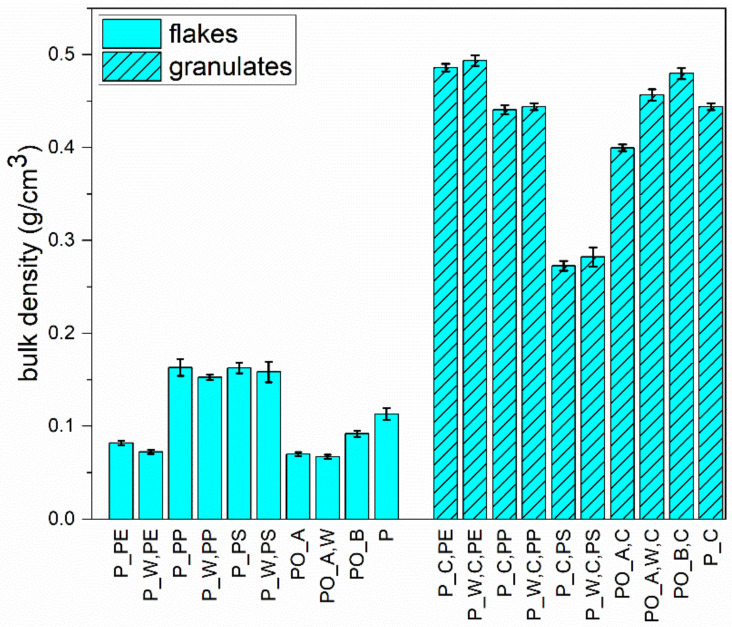
Determined bulk densities of all investigated materials before (flakes <4 mm) and after homogenisation (granulates).

**Figure A5:**
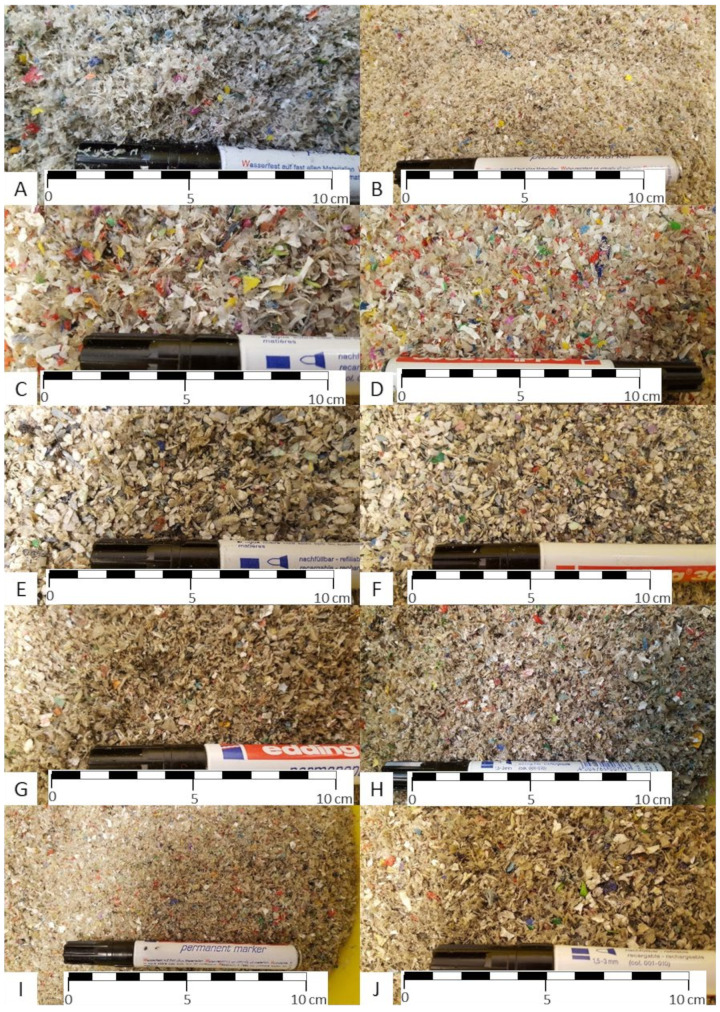
Exemplary photos of the flakes of all investigated materials after shredding to <4 mm: P_PE—**A**; P_W,PE—**B**; P_PP—**C**; P_W,PP—**D**; P_PS—**E**; P_W,PS—**F**; PO_A—**G**; PO_W,**A–H**; PO_B—**I**; and P—**J**.

**Figure A6:**
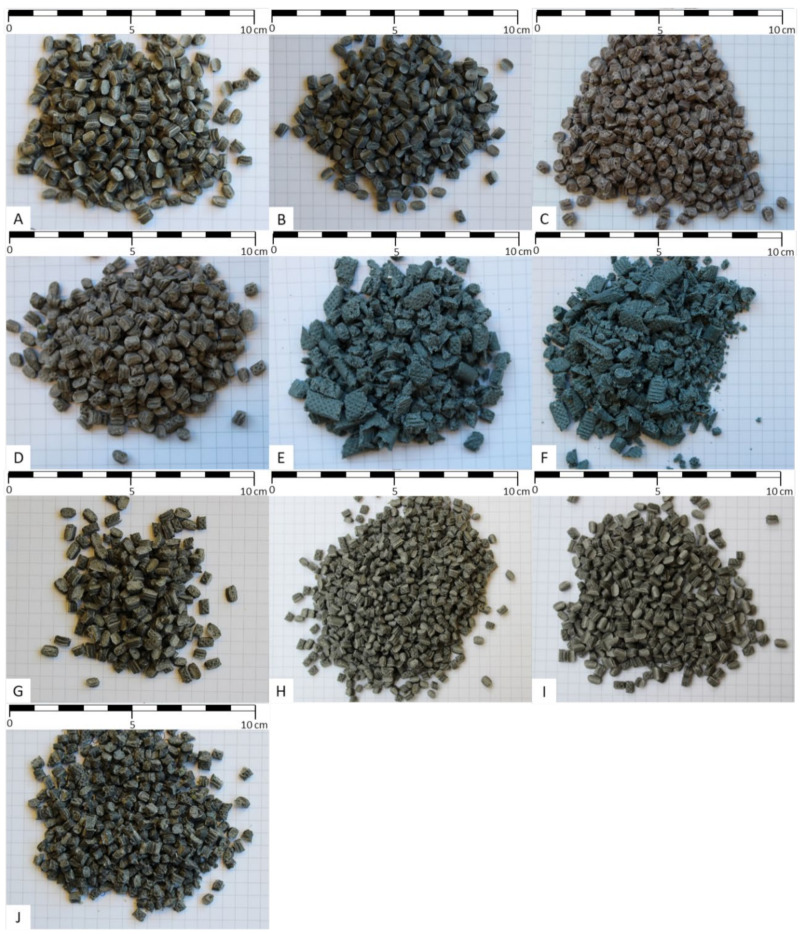
Exemplary photos of the granulates of all investigated materials after homogenisation and pelletising: P_C,PE—**A**; P_W,C,PE—**B**; P_PP_C—**C**; P_W,C,PP—**D**; P_C,PS—**E**; P_W,C,PS—**F**; PO_A,C—**G**; PO_W,**C**,**A–H**; PO_B,**C—I**; and P_C—**J**.

**Figure A7:**
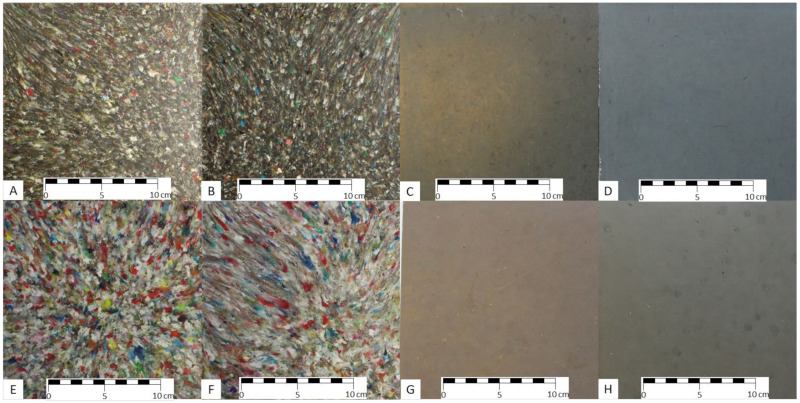
Exemplary photos of vacuum compression moulded plates: P_PE—**A**; P_W,PE—**B**; P_C,PE—**C**; P_W,C,PE—**D**; P_PP—**E**; P_W,PP—**F**; P_C,PP—**G**; and P_W,C,PP—**H**.

**Figure A8:**
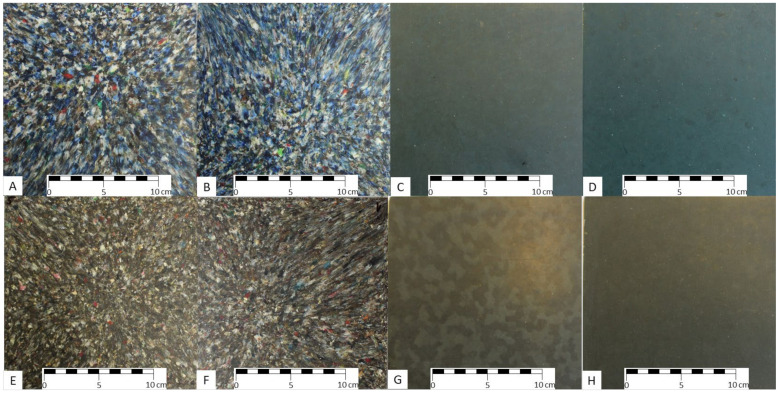
Exemplary photos of vacuum compression moulded plates: P_PS—**A**; P_W,PS—**B**; P_C,PS—**C**; P_W,C,PS—**D;** PO_A—**E**; PO_A,W—**F**; PO_A,**C**–**G**; and PO_A,W,**C**–**H**.

**Figure A9:**
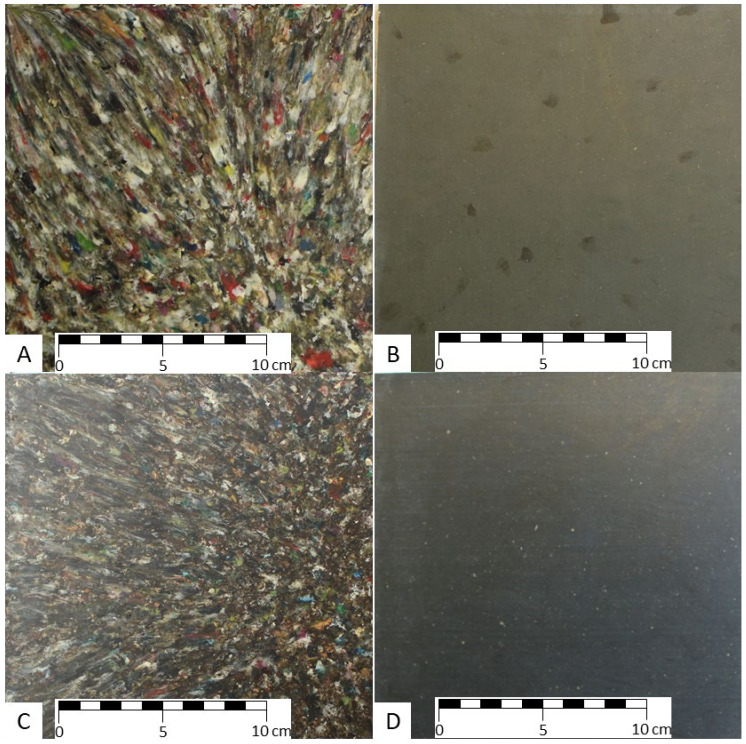
Exemplary photos of vacuum compression moulded plates: PO_B—**A**; PO_B,W—**B**; P—**C**; and P_C—**D**.

The determined bulk densities of all materials are shown in Figure 5 before (flakes <4 mm) and after homogenisation (granulates). The results of the bulk densities of all investigated materials are given in Appendix G. Exemplary photos of the produced flakes (Figure A5), and granulates (Figure A6) are given in Appendix I.

#### 3.6.1. Flakes

As expected, the bulk densities of all flakes are lower than those of the granules. The PE flakes have a bulk density of 0.082 ± 0.0023 g/cm^3^ (P_PE) and 0.072 ± 0.0022 g/cm^3^ (P_W,PE). The PP and PS flakes have a bulk density of approx. 0.16 g/cm^3^, almost twice as high. For PP, this can be explained by the higher proportion of compacted, three-dimensional particles, although vPP (0.895–0.91 g/cm^3^) is in the same material density range as vPE (0.87–0.97 g/cm^3^) [24]. The PE flakes consist mainly of flat, thin, two-dimensional particles, although vPS has a higher material density (0.104–0.109 g/cm^3^) [24] than vPE or vPP, and 2D particles are less common. Nevertheless, PS can occur in an expanded form (EPS: 0.0015–0.009 g/cm^3^) [31], which would significantly reduce the bulk density. The PO_A flakes have a bulk density of about 0.07 g/cm^3^. This suggests that the flakes contain a high proportion of mainly PE films. The bulk density of PO_B is 0.092 g/cm^3^ and of P is 0.113 g/cm^3^. No feeding problems (e.g., bridge formation) were observed for the flakes of all materials.

#### 3.6.2. Granulates

The homogeneous PE granulates have the highest bulk density with 0.49 g/cm^3^. The PP granulates have a bulk density of 0.44 g/cm^3^. The PS granulates have the lowest bulk density of the homogeneous materials with approx. 0.27 g/cm^3^. This can be explained by the fact that degradation processes caused increased outgassing during extrusion, which could not be sufficiently removed (see Figure A6 in Appendix I). Additionally, the different bulk densities of PO_A,C (0.4 g/cm^3^) and PO_A,W,C (0.46 g/cm^3^) can be explained in this way. The bulk density of PO_B,C was the second highest with 0.48 g/cm^3^. P_C had a bulk density of 0.44 g/cm^3^. Commercially available plastic granulates have a bulk density between 0.5 and 0.9 g/cm^3^ [32]. No feeding problems (e.g., bridge formation) were observed for the granulates produced from all materials.

## 4. Discussion

The degree of crystallinity of polymers is directly related to their material properties: the more crystalline a polymer is, the harder and more brittle it is, and dimensional stability and melting point or softening point increase because intermolecular forces can act more effectively due to the more uniform arrangement of the molecules [13,22]. Despite the low crystallinity (36–38%), the PE materials examined, except for P_W,C,PE, show predominantly brittle material behaviour. Since a washing process somewhat improves the mechanical properties, it can be concluded that these are mainly impurities that negatively influence the material properties and that material ageing plays a subordinate role. Likewise, the distinct, second melting temperature at about 128 °C for P_PP and P_C,PP can be explained by the presence of organic impurities (e.g., other plastics with a density >1 g/cm^3^), which can also be removed by washing. Therefore, T_C_ and T_m1_ of the PS materials can only be explained by contained organic impurities, e.g., PP.

The DSC curve progressions of the investigated PO materials as well as the determined values of T_C_ and a distinct T_m1_ at approx. 125 °C and a T_m2_ at approx. 163 °C indicate a higher PE than PP content of the PO materials. The melting temperature at approx. 110 °C of some PO materials and P_W,PE can be attributed to organic impurities or a higher LDPE content. A second crystallisation peak at approx. 120 °C for PO_B,C becomes clear from the HDPE content [34]. This and the higher ΔH_m_ of T_m2_ allow the assumption of a somewhat higher PP content than in PO_A. The similar DSC curves of mixed plastics (P) to the investigated PO materials lead to the hypothesis that most investigated P materials consist of PO. This indicates that a separation, and separate PE, PP, and PO processing is not necessary since the thermal properties do not change significantly.

The MFR results are surprisingly good for almost all materials examined, which indicate low material damage and thus, good processability. Depending on the material (high or low viscosity), suitable processing methods must be selected. The investigated PO and P materials have similar MRF values to the investigated PE materials. From this, it can be concluded for the MFR that a separation of the PE materials out of mixed plastics is not mandatory.

Except for the very brittle PS materials, all other materials investigated have surprisingly good mechanical properties. The mechanical characteristics show that wet processing, combined with a homogenisation step, does not necessarily lead to an improvement in mechanical properties. Most of the investigated materials show a clear decrease in mechanical properties compared to virgin homopolymers known from the literature. This indicates the existence of organic and inorganic impurities as well as material degradation due to ageing [35].

The investigations on the ash content of the materials show high inorganic contents, which are mostly significantly reduced by washing. These inorganic impurities are a plausible explanation for the observed deviations between the washed and unwashed materials. Additionally, a short service life (<1 year) is to be expected for the plastics in the wastes examined. It is therefore assumed that material ageing plays only a minor role. A part of the AC is due to inorganic fillers (e.g., glass fibres, silicates, oxides, and hydroxides) in the polymer matrix. Thus, despite the comparatively high ash contents (3–8%), the PO and P materials investigated show a good mechanical property profile. This suggests that the inorganic impurities contained act to a certain extent as a reinforcing material.

Knowledge of the bulk density of free-flowing materials is an essential parameter for the design of storage, transport, and dosing equipment. The bulk density is also essential for the material feed behaviour and the pressure build-up in solid conveying areas of extruders or injection moulding machines [36]. It should be noted that the pelletising system’s settings and the melt strand temperature have a significant influence on the pellet geometry and, thus, on the bulk density [31]. Commercially available plastic granulates have a bulk density between 0.5 and 0.9 g/cm^3^ [33]. As the granulate bulk densities determined are only slightly lower, with the exception of P_C,PS, and P_W,C,PS, it is assumed that these materials have good conveying and feeding properties. No feeding problems (e.g., bridging) occurred with the flakes and granulates produced from all materials.

## 5. Conclusions

The investigations have shown that all waste materials could be processed into plastic-rich fractions with a grain size < 4 mm using simple waste treatment without any significant problems. The processing of the different plastic fractions with simple compression moulding showed that all PE, PP, PO, and P materials could be processed without any major problems. This, together with the low MFR values, suggests that conventional extrusion into semi-finished products such as pipes or plates could be technically possible. A list of potential products for the materials investigated is given in Appendix J. The injection moulding process must be tested, and investigations must be carried out with a high-pressure capillary rheometer. Furthermore, thermogravimetric (TGA) and Fourier-transform infrared (FTIR) analysis to determine chemical structure changes possible for polymeric waste during the technological process of the materials is recommended for future investigations. The PS materials emitted gas in both processing variants, and several test runs were necessary to find a stable processing method. Therefore, an evaluation of volatile organic compound emissions from the materials is necessary if they would be implemented in industrial processes. The PET materials could neither be homogenised nor compression moulded due to excessive contamination.

In some cases, the material properties determined are (Young’s modulus, impact strength) clearly below those of virgin polymers. This limits the product range that can be manufactured and its range of applications. The results also show that an upstream washing process improves the achievable properties, but homogenisation does not necessarily improve properties. It was also found that a higher treatment depth (recovery of plastic types) from mixed wastes is not necessary since the PO and mixed plastics fractions showed similarly good material data with good processability.

In summary, the investigations show that the recovery and simple treatment of plastics from mixed, contaminated wastes into at least downcycling products seems to be possible. The transfer of used plastics from thermal recovery to recycling could make an important contribution to achieving additional recycling targets, resource conservation, and CO_2_ and waste reduction.

## Figures and Tables

**Table 1:** Results of the DSC measurements: crystallisation temperature (T_C_), crystallisation enthalpy (ΔH_c_), melting temperatures (T_m1_ and T_m2_), melting enthalpy (ΔH_m1_ and ΔH_m2_), and glass transition temperature (T_g_).

Parameters	T_C_	ΔH_c_	T_m1_	ΔH_m1_	T_m2_	ΔH_m2_	T_g_
Material	(°C)	(J/g)	(°C)	(J/g)	(°C)	(J/g)	(°C)
P_PE	110.3 ± 2.2	115.8 ± 18.6	129.3 ± 2.8	94.4 ± 18.6	162.8 ± 1.2	4.8 ± 4	−
P_W,PE	105.1 ± 1.8	118.9 ± 8.2	125.4 ± 0.7	97.1 ± 10.4	161.5 ± 0.6	4 ± 1.4	−
P_C,PE	107.6 ± 0.3	109.7 ± 4.8	127.6 ± 0.2	80.9 ± 2.3	162.7 ± 0.1	4.5 ± 0.2	−
P_W,C,PE	108.8 ± 1.3	128.3 ± 3	127.1 ± 0.8	98.7 ± 1.8	−	−	−
P_PP	116 ± 2.9	86.7 ± 8.8	165.3 ± 0.8	74.2 ± 11.2	−	−	−
P_W,PP	115.5 ± 2.5	91.8 ± 2.7	166.3 ± 1	79.6 ± 6.9	−	−	−
P_C,PP	119.4 ± 1.6	83.5 ± 4.1	164 ± 1	47.6 ± 18.3	128.1 ± 0.6	6.1±0.2	−
P_W,C,PP	119 ± 0.8	83.5 ± 2.1	163.8 ± 0.7	63.6 ± 0.5	−	−	−
P_PS	119.6 ± 3.9	10.4 ± 8.9	163.5 ± 1.4	7.7 ± 9.5	−	−	99.5 ± 0.9
P_W,PS	113.4 ± 3.5	5.2 ± 2.4	162.2 ± 0.9	2.8 ± 2.2	−	−	98.9 ± 1.6
P_C,PS	114 ± 0.3	7.7 ± 0.9	161.5 ± 0.1	4.5 ± 0.3	−	−	98.3 ± 0.2
P_W,C,PS	−	−	161.9 ± 0.1	3.6 ± 0.2	−	−	98.9 ± 0.4
PO_A	108.7 ± 4.6	74.1 ± 18.9	124.2 ± 1.2	46.1 ± 14.9	163.1 ± 1.1	13.7 ± 4.2	−
PO_A,W	107.9 ± 2.4	81.1 ± 4.6	125.4 ± 0.8	50.8 ± 4.8	163.5 ± 1.3	15 ± 4.6	−
PO_A,C	108.3 ± 1.1	83.8 ± 0.3	125.4 ± 0.3	50.2 ± 2.9	162 ± 0.5	15.1 ± 0.2	−
PO_A,W,C	107.8 ± 1.5	86.6 ± 3.2	125.4 ± 0.6	51 ± 0.5	162 ± 0.7	17.5 ± 1.4	−
PO_B	110.4 ± 3.9	105 ± 10.3	129 ± 4.9	54.6 ± 12.4	163.7 ± 1	18.8 ± 3.9	−
PO_B,C	110.4 ± 1.4	110 ± 1.5	126.7 ± 0.3	61.9 ± 3.1	162 ± 0.6	22.1 ± 0.9	−
P	111.2 ± 1.8	52.2 ± 10.6	128.2 ± 6.9	31.5 ± 9.4	164.2 ± 1.2	12 ± 7.1	−
P_C	110.6 ± 0.5	67.3 ± 2.4	126.4 ± 0.3	39.6 ± 3.1	161.1 ± 0.4	13.1 ± 0.3	−

**Figure A1:**
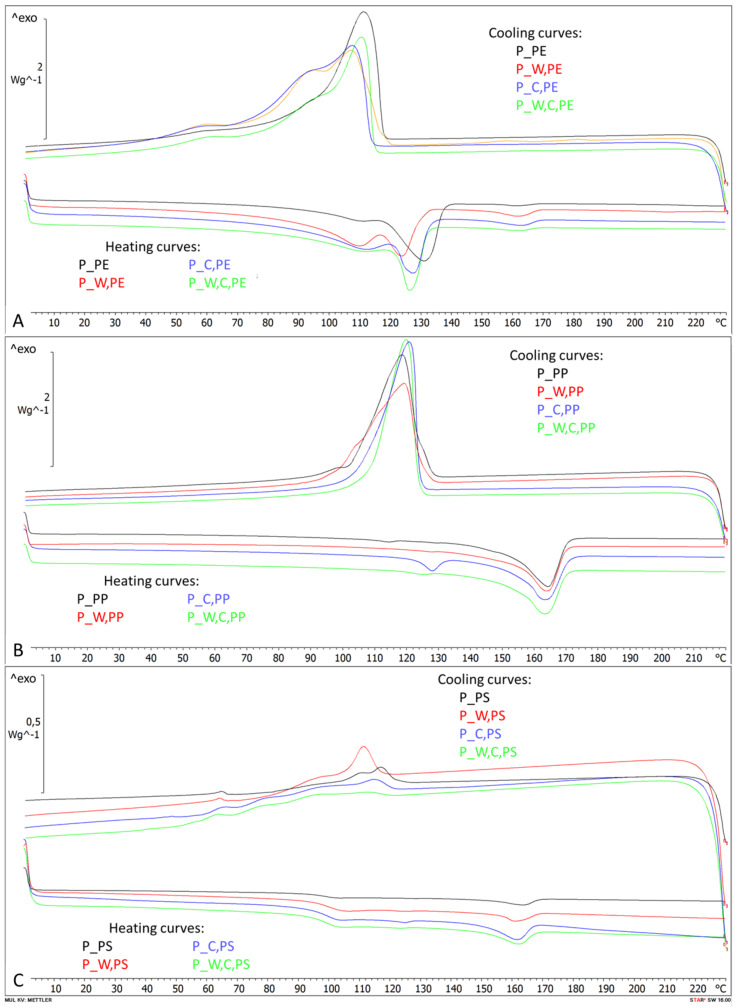

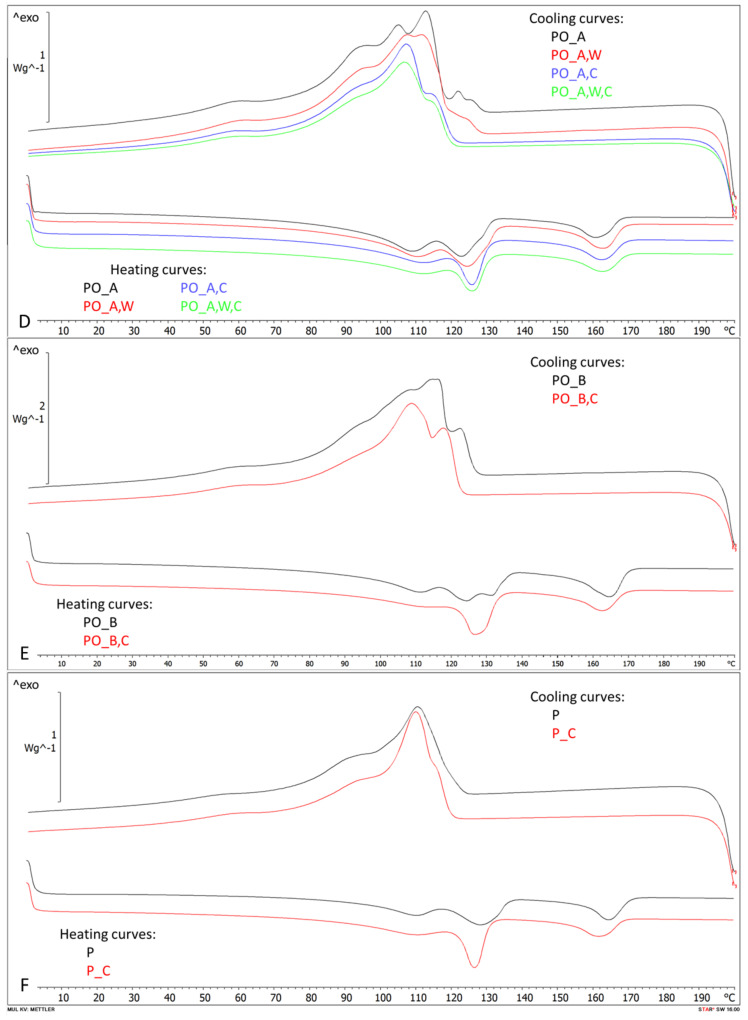
Representative DSC measurement results for (**A**) PE, (**B**) PP, (**C**) PS materials. Note: cooling with 20 K/min on top and second heating with 10 K/min at the bottom of each diagram, (**D**) PO_A, (**E**) PO_B, and (**F**) P materials. Note: cooling with 20 K/min on top and second heating with 10 K/min at the bottom of each diagram.

## Data Availability

Not applicable.

## Abbreviations

**Abbreviation**	**Description**
ΔH_C_	crystallisation enthalpy
ΔH_m_	melting enthalpy
ε_B_	elongation at break
ε_M_	maximum elongation at the yield point
σ_B_	tensile strength at break
σ_M_	maximum tensile strength
AC	ash content
a_cN_	notched impact strength
a_cU_	impact strength
C	homogenised
CO_2_	carbon dioxide—greenhouse gas
D	screw diameter
DS	dry substance
DSC	differential scanning calorimetry
E	Young’s modulus
e.g.,	for example
EPS	expanded polystyrene
EU	European Union
FTIR	Fourier-transform infrared
HDPE	high-density polyethylene
LDPE	low-density polyethylene
LLDPE	linear low-density polyethylene
min	minutes
MFR	melt (mass) flow rate
Mt	million tonnes
NIR	near-infrared
OS	original substance
P	plastics
P&C	paper and cardboard
PO	polyolefins
(v)PE	(virgin) polyethylene
PET	polyethylene terephthalate
PP	polypropylene
PS	polystyrene
PVC	polyvinyl chloride
rpm	revolutions per minute
SRF	solid recovered fuel
T_C_	crystallisation temperature
Tg	glass transition temperature
T_m_	melting temperature
TSE	twin-screw extruder
v	virgin
W	washed

## Appendix A. Total Composition of SRF and Plastic Type Content

**Table A1:** Total composition of SRF determined by manual sorting analysis (Note: fine fraction <20 (8.5%) mm was separated and is not considered in the table).

Fraction	Mass (kg)	Mass (%)
Plastics	150.4	86.5
Metals	1.01	0.6
P&C ^1^	10.03	5.8
Inert	1.04	0.6
Wood	0.17	0.1
Other	11.27	6.5
**Total**	173.92	100

^1^ P&C: paper and cardboard.

**Table A2:** Plastic type content of P determined by sensor-based sorting with near-infrared.

Fraction	Mass (kg)	Mass (%)
PE	44.64	36.42
PP	19.19	15.66
PET	25.34	20.67
PS	7.41	6.04
Other	26	21.21
**Total**	122.58	100

## Appendix C. Processing Conditions

**Table A3:** Extrusion conditions for homogenization of all investigated materials.

Materials:	P_PE; P_W,PE; P_PP; P_W,PP; P_PS; P_W,PS			
Zones	Zone 1	Zone 2	Zone 3	Zone 4
Temperature (°C)	150	170	170	170
Materials:	PO_A; PO_A,W; PO_B			
	Zone 1	Zone 2	Zone 3	Zone 4
Temperature (°C)	150	180	200	205

**Table A4:** Compression moulding conditions of all investigated materials.

**Materials:**	**P_PE**	**P_W,PE**			
**Phases**	Phase 1	Phase 2	Phase 3	Phase 4	Phase 5
Temperature (°C)	210	210	210	210	30
Pressure (bar)	1	10	50	100	100
Time (min)	8	5	4	4	15
Materials:	P_C,PE	P_W,C,PE			
**Phases**	Phase 1	Phase 2	Phase 3	Phase 4	Phase 5
Temperature (°C)	200	200	200	200	30
Pressure (bar)	1	10	50	100	100
Time (min)	10	5	4	4	15
Materials:	P_PP	P_W,PP	P_C,PP	P_W,C,PP	
**Phases**	Phase 1	Phase 2	Phase 3	Phase 4	Phase 5
Temperature (°C)	200	200	200	200	30
Pressure (bar)	1	10	50	100	100
Time (min)	14	5	4	4	15
Materials:	P_PS	P_W,PS	P_C,PS	P_W,C,PS	
**Phases**	Phase 1	Phase 2	Phase 3	Phase 4	Phase 5
Temperature (°C)	207	205	205	205	30
Pressure (bar)	5	10	50	100	100
Time (min)	10	5	4	4	15
Materials: PO_A	PO_A,W	PO_A,C	PO_A,W,C	PO_B	PO_B,C
**Phases**	Phase 1	Phase 2	Phase 3	Phase 4	Phase 5
Temperature (°C)	210	210	210	210	30
Pressure (bar)	1	10	50	100	100
Time (min)	8	5	4	4	15
Materials:	P	P_C			
**Phases**	Phase 1	Phase 2	Phase 3	Phase 4	Phase 5
Temperature (°C)	210	210	210	210	30
Pressure (bar)	1	10	50	100	100
Time (min)	8	5	4	4	15

## Appendix D. MFR Measurements

**Table A5:** MFR measurement results.

Sample Identification	Sample Number	Total Mass (g)	Time Interval (min)	MFR (g/10 min)	Mean Value (g/10 min)	Standard Deviation (g/10 min)
P_PE	P1	2.10	10	2.571	2.479	0.130
P_PE	P2	2.09	10	2.387		
P_W,PE	P1	2.71	10	2.586	2.641	0.078
P_W,PE	P2	3.08	10	2.696		
P_C,PE	P1	2.38	10	2.184	2.147	0.053
P_C,PE	P2	2.31	15	2.110		
P_W,C,PE	P1	2.41	20	1.794	1.820	0.037
P_W,C,PE	P2	2.40	20	1.846		
P_PP	P1	2.20	5	1.805	1.304	0.708
P_PP	P2	2.18	10	0.803		
P_W,PP	P1	2.78	10	11.801	12.286	0.687
P_W,PP	P2	3.35	5	12.772		
P_C,PP	P1	2.53	10	3.932	4.178	0.130
P_C,PP	P2	2.62	10	4.029		
P_W,C,PP	P1	2.31	10	7.114	7.273	0.225
P_W,C,PP	P2	3.19	10	7.432		
P_PS	P1	2.82	20	0.218	0.927	1.003
P_PS	P2	2.56	10	1.636		
P_W,PS	P1	3.21	20	1.744	1.217	0.746
P_W,PS	P2	2.94	20	0.689		
P_C,PS	P1	2.00	10	5.673	5.026	0.914
P_C,PS	P2	2.00	10	4.380		
P_W,C,PS	P1	2.78	10	3.526	2.891	0.898
P_W,C,PS	P2	2.73	10	2.255		
PO_A	P1	2.67	20	0.936	0.920	0.023
PO_A	P2	2.72	20	0.904		
PO_A,W	P1	2.56	20	0.410	0.855	0.630
PO_A,W	P2	2.50	20	1.301		
PO_A,C	P1	2.23	20	1.934	2.042	0.153
PO_A,C	P2	2.56	20	2.150		
PO_A,W,C	P1	2.34	15	2.220	2.280	0.085
PO_A,W,C	P2	2.71	15	2.341		
PO_B	P1	2.40	10	3.012	3.338	0.460
PO_B	P2	2.44	10	3.663		
PO_B,C	P1	2.64	10	2.690	2.700	0.013
PO_B,C	P2	2.39	10	2.709		
P	P1	2.32	10	2.798	2.319	0.677
P	P2	2.94	10	1.840		
P_C	P1	2.35	10	3.333	3.676	0.485
P_C	P2	2.50	10	4.019		

## Appendix E. Impact Tests

**Table A6:** Results of the notched impact tests and unnotched impact tests of P_PE.

Sample Number	l (mm)	b_B_ (mm)	d (mm)	A (m^2^)	W (J)	W_empty_ (J)	W_corr_ (kJ)	a_cN_ (kJ/m^2^)	Note
P1	78.54	10.10	3.51	0.000035	0.988	0.004	0.000984	27.8	partially broken
P2	78.35	10.09	3.40	0.000034	0.500	0.004	0.000496	14.5	partially broken
P3	78.50	10.07	3.42	0.000034	0.596	0.004	0.000592	17.2	partially broken
P4	78.62	10.09	3.42	0.000035	0.452	0.004	0.000448	13.0	
P5	78.26	10.08	3.48	0.000035	0.660	0.004	0.000656	18.7	
P6	78.62	8.12	3.51	0.000029	0.322	0.007	0.000315	11.1	
P7	78.64	8.15	3.51	0.000029	0.298	0.007	0.000291	10.2	
P8	78.72	7.89	3.48	0.000027	0.357	0.007	0.000350	12.7	
P9	78.55	8.00	3.53	0.000028	0.324	0.007	0.000317	11.2	
P10	78.71	7.95	3.49	0.000028	0.255	0.007	0.000248	8.9	

**Table A7:** Results of the notched impact tests and unnotched impact tests of P_W,PE.

Sample Number	l (mm)	b_B_ (mm)	d (mm)	A (m^2^)	W (J)	W_empty_ (J)	W_corr_ (kJ)	a_cN_ (kJ/m^2^)	Note
P1	78.94	10.08	3.50	0.000035	0.808	0.004	0.000804	22.8	partially broken
P2	79.04	10.04	3.52	0.000035	0.732	0.004	0.000728	20.6	partially broken
P3	79.03	9.95	3.49	0.000035	0.604	0.004	0.000600	17.3	partially broken
P4	78.58	10.03	3.51	0.000035	0.844	0.004	0.000840	23.9	partially broken
P5	79.10	10.15	3.51	0.000036	0.772	0.004	0.000768	21.6	partially broken
P6	77.55	7.90	3.70	0.000029	0.348	0.007	0.000341	11.7	
P7	78.45	7.94	3.53	0.000028	0.337	0.007	0.000330	11.8	
P8	78.46	7.84	3.54	0.000028	0.345	0.007	0.000338	12.2	partially broken
P9	78.30	8.03	3.49	0.000028	0.348	0.007	0.000341	12.2	
P10	77.60	7.79	3.60	0.000028	0.364	0.007	0.000357	12.7	partially broken

**Table A8:** Results of the notched impact tests and unnotched impact tests of P_C,PE.

Scheme Number	l (mm)	b_B_ (mm)	d (mm)	A (m^2^)	W (J)	W_empty_ (J)	W_corr_ (kJ)	a_cN_ (kJ/m^2^)	Note
P1	79.17	10.14	3.69	0.000037	0.764	0.004	0.000760	20.3	
P2	79.06	10.15	3.72	0.000038	1.068	0.004	0.001064	28.2	
P3	79.17	10.18	3.75	0.000038	0.604	0.004	0.000600	15.7	
P4	79.03	10.17	3.65	0.000037	0.716	0.004	0.000712	19.2	
P5	78.08	10.14	3.64	0.000037	1.240	0.004	0.001236	33.5	
P6	78.13	7.96	3.58	0.000028	0.213	0.007	0.000206	7.2	
P7	78.04	7.95	3.55	0.000028	0.213	0.007	0.000206	7.3	
P8	78.03	8.01	3.56	0.000029	0.230	0.007	0.000223	7.8	
P9	78.10	8.00	3.68	0.000029	0.206	0.007	0.000199	6.8	
P10	78.10	8.01	3.61	0.000029	0.214	0.007	0.000207	7.2	

**Table A9:** Results of the notched impact tests and unnotched impact tests of P_W,C,PE.

Sample Number	l (mm)	b_B_ (mm)	d (mm)	A (m^2^)	W (J)	W_empty_ (J)	W_corr_ (kJ)	a_cN_ (kJ/m^2^)	Note
P1	78.52	10.18	3.59	0.000037	2.080	0.030	0.002050	56.1	not broken
P2	78.18	10.16	3.54	0.000036	1.260	0.030	0.001230	34.2	not broken
P3	78.37	10.19	3.53	0.000036	3.250	0.030	0.003220	89.5	not broken
P4	78.25	10.22	3.55	0.000036	2.770	0.030	0.002740	75.5	not broken
P5	78.49	10.15	3.53	0.000036	3.000	0.030	0.002970	82.9	not broken
P6	78.67	8.00	3.57	0.000029	0.270	0.007	0.000263	9.2	
P7	78.72	7.90	3.58	0.000028	0.251	0.007	0.000244	8.6	
P8	78.77	7.82	3.56	0.000028	0.255	0.007	0.000248	8.9	
P9	78.74	8.05	3.60	0.000029	0.267	0.007	0.000260	9.0	
P10	78.79	8.06	3.59	0.000029	0.252	0.007	0.000245	8.5	

**Table A10:** Results of the notched impact tests and unnotched impact tests of P_PP.

Sample Number	l (mm)	b_B_ (mm)	d (mm)	A (m^2^)	W (J)	W_empty_ (J)	W_corr_ (kJ)	a_cN_ (kJ/m^2^)	Note
P1	78.60	10.24	3.81	0.000039	0.188	0.008	0.000180	4.6	
P2	77.92	10.25	3.62	0.000037	0.258	0.008	0.000250	6.7	
P3	77.94	10.25	3.62	0.000037	0.126	0.008	0.000118	3.2	
P4	77.92	10.25	3.63	0.000037	0.152	0.008	0.000144	3.9	
P5	77.91	10.25	3.62	0.000037	0.213	0.008	0.000205	5.5	
P6	78.80	8.37	3.59	0.000030	0.084	0.007	0.000077	2.6	
P7	79.26	8.29	3.63	0.000030	0.073	0.007	0.000066	2.2	
P8	78.20	8.20	3.60	0.000030	0.097	0.007	0.000090	3.0	
P9	78.06	8.13	3.63	0.000030	0.095	0.007	0.000088	3.0	
P10	77.68	8.57	3.67	0.000031	0.069	0.007	0.000062	2.0	

**Table A11:** Results of the notched impact tests and unnotched impact tests of P_W,PP.

Sample Number	l (mm)	b_B_ (mm)	d (mm)	A (m^2^)	W (J)	W_empty_ (J)	W_corr_ (kJ)	a_cN_ (kJ/m^2^)	Note
P1	77.24	10.25	3.65	0.000037	0.220	0.004	0.000216	5.8	
P2	77.65	10.30	3.60	0.000037	0.180	0.004	0.000176	4.7	
P3	77.40	10.24	3.58	0.000037	0.312	0.004	0.000308	8.4	
P4	77.88	10.18	3.60	0.000037	0.216	0.004	0.000212	5.8	
P5	77.59	10.20	3.55	0.000036	0.264	0.004	0.000260	7.2	
P6	79.02	7.96	3.65	0.000029	0.068	0.007	0.000061	2.1	
P7	77.68	7.96	3.80	0.000030	0.064	0.007	0.000057	1.9	
P8	77.86	8.38	3.76	0.000032	0.106	0.007	0.000099	3.1	
P9	77.92	8.01	3.77	0.000030	0.072	0.007	0.000065	2.2	
P10	79.02	8.22	3.80	0.000031	0.096	0.007	0.000089	2.8	

**Table A12:** Results of the notched impact tests and unnotched impact tests of P_C,PP.

Sample Number	l (mm)	b_B_ (mm)	d (mm)	A (m^2^)	W (J)	W_empty_ (J)	W_corr_ (kJ)	a_cN_ (kJ/m^2^)	Note
P1	77.61	10.35	3.88	0.000040	0.197	0.008	0.000189	4.7	
P2	78.07	10.25	3.70	0.000038	0.221	0.008	0.000213	5.6	
P3	77.84	10.10	3.70	0.000037	0.357	0.008	0.000349	9.3	
P4	78.05	10.22	3.73	0.000038	0.363	0.008	0.000355	9.3	
P5	77.47	10.02	3.69	0.000037	0.382	0.008	0.000374	10.1	
P6	77.77	8.06	3.68	0.000030	0.121	0.007	0.000114	3.8	
P7	78.08	8.24	3.64	0.000030	0.116	0.007	0.000109	3.6	
P8	77.89	8.37	3.66	0.000031	0.106	0.007	0.000099	3.2	
P9	77.85	8.14	3.69	0.000030	0.108	0.007	0.000101	3.4	
P10	77.94	8.43	3.92	0.000033	0.119	0.007	0.000112	3.4	

**Table A13:** Results of the notched impact tests and unnotched impact tests of P_W,C,PP.

Sample Number	l (mm)	b_B_ (mm)	d (mm)	A (m^2^)	W (J)	W_empty_ (J)	W_corr_ (kJ)	a_cN_ (kJ/m^2^)	Note
P1	77.87	10.25	3.70	0.000038	0.948	0.004	0.000944	24.9	
P2	77.85	10.20	3.71	0.000038	0.464	0.004	0.000460	12.2	
P3	77.49	10.25	3.80	0.000039	0.508	0.004	0.000504	12.9	
P4	77.50	10.25	3.63	0.000037	0.244	0.004	0.000240	6.5	
P5	77.47	10.00	3.65	0.000037	0.412	0.004	0.000408	11.2	
P6	77.74	8.14	3.65	0.000030	0.131	0.007	0.000124	4.2	
P7	77.68	8.02	3.65	0.000029	0.133	0.007	0.000126	4.3	
P8	77.74	8.22	3.65	0.000030	0.113	0.007	0.000106	3.5	
P9	77.78	7.99	3.67	0.000029	0.139	0.007	0.000132	4.5	
P10	77.63	8.28	3.61	0.000030	0.147	0.007	0.000140	4.7	

**Table A14:** Results of the notched impact tests and unnotched impact tests of P_PS.

Sample Number	l (mm)	b_B_ (mm)	d (mm)	A (m^2^)	W (J)	W_empty_ (J)	W_corr_ (kJ)	a_cN_ (kJ/m^2^)	Note
P1	79.32	10.30	3.88	0.000040	0.121	0.006	0.000115	2.9	
P2	79.54	10.30	3.86	0.000040	0.098	0.006	0.000092	2.3	
P3	79.24	10.30	3.85	0.000040	0.129	0.006	0.000123	3.1	
P4	79.75	10.30	3.91	0.000040	0.143	0.006	0.000137	3.4	
P5	79.59	10.10	3.90	0.000039	0.149	0.006	0.000143	3.6	
P6	79.10	8.03	3.93	0.000032	0.091	0.006	0.000085	2.7	
P7	78.58	7.93	3.98	0.000032	0.085	0.006	0.000079	2.5	
P8	78.46	7.83	3.96	0.000031	0.083	0.006	0.000077	2.5	
P9	78.62	7.95	3.98	0.000032	0.085	0.006	0.000079	2.5	
P10	78.78	8.01	3.95	0.000032	0.088	0.006	0.000082	2.6	

**Table A15:** Results of the notched impact tests and unnotched impact tests of P_W,PS.

Sample Number	l (mm)	b_B_ (mm)	d (mm)	A (m^2^)	W (J)	W_empty_ (J)	W_corr_ (kJ)	a_cN_ (kJ/m^2^)	Note
P1	79.61	10.29	3.89	0.000040	0.101	0.008	0.000093	2.3	
P2	79.39	10.18	3.88	0.000039	0.152	0.008	0.000144	3.6	
P3	80.18	10.21	3.85	0.000039	0.109	0.008	0.000101	2.6	
P4	79.55	10.27	3.95	0.000041	0.145	0.008	0.000137	3.4	
P5	79.47	10.35	3.87	0.000040	0.157	0.008	0.000149	3.7	
P6	78.44	7.88	3.89	0.000031	0.110	0.007	0.000103	3.4	
P7	78.31	8.17	3.93	0.000032	0.109	0.007	0.000102	3.2	
P8	79.49	8.39	3.84	0.000032	0.092	0.007	0.000085	2.6	
P9	79.65	8.18	3.85	0.000031	0.079	0.007	0.000072	2.3	
P10	79.74	8.50	3.86	0.000033	0.091	0.007	0.000084	2.6	

**Table A16:** Results of the notched impact tests and unnotched impact tests of P_C,PS.

Sample Number	l (mm)	b_B_ (mm)	d (mm)	A (m^2^)	W (J)	W_empty_ (J)	W_corr_ (kJ)	a_cN_ (kJ/m^2^)	Note
P1	79.92	10.40	3.99	0.000041	0.157	0.008	0.000149	3.6	
P2	79.79	10.40	4.03	0.000042	0.230	0.008	0.000222	5.3	
P3	78.26	10.36	3.95	0.000041	0.175	0.008	0.000167	4.1	
P4	79.78	10.40	4.02	0.000042	0.157	0.008	0.000149	3.6	
P5	79.92	10.14	3.99	0.000040	0.222	0.008	0.000214	5.3	
P6	78.64	8.26	3.97	0.000033	0.050	0.007	0.000043	1.3	
P7	78.68	8.12	3.96	0.000032	0.048	0.007	0.000041	1.3	
P8	78.73	8.07	3.98	0.000032	0.047	0.007	0.000040	1.2	
P9	78.72	8.01	3.99	0.000032	0.052	0.007	0.000045	1.4	
P10	78.85	7.95	4.02	0.000032	0.047	0.007	0.000040	1.3	

**Table A17:** Results of the notched impact tests and unnotched impact tests of P_W,C,PS.

Sample Number	l (mm)	b_B_ (mm)	d (mm)	A (m^2^)	W (J)	W_empty_ (J)	W_corr_ (kJ)	a_cN_ (kJ/m^2^)	Note
P1	79.42	10.30	4.04	0.000042	0.155	0.008	0.000147	3.5	
P2	79.09	10.25	4.00	0.000041	0.114	0.008	0.000106	2.6	
P3	79.28	10.35	3.95	0.000041	0.163	0.008	0.000155	3.8	
P4	79.44	10.30	3.91	0.000040	0.179	0.008	0.000171	4.2	
P5	79.45	10.31	3.90	0.000040	0.217	0.008	0.000209	5.2	
P6	79.08	8.02	3.94	0.000032	0.067	0.007	0.000060	1.9	
P7	79.12	8.04	3.87	0.000031	0.055	0.007	0.000048	1.5	
P8	79.36	8.08	3.99	0.000032	0.054	0.007	0.000047	1.5	
P9	79.29	7.88	3.98	0.000031	0.049	0.007	0.000042	1.3	
P10	79.37	8.20	3.89	0.000032	0.055	0.007	0.000048	1.5	

**Table A18:** Results of the notched impact tests and unnotched impact tests of PO_A.

Sample Number	l (mm)	b_B_ (mm)	d (mm)	A (m^2^)	W (J)	W_empty_ (J)	W_corr_ (kJ)	a_cN_ (kJ/m^2^)	Note
P1	79.36	10.03	3.76	0.000038	0.276	0.008	0.000268	7.1	partially broken
P2	79.57	10.06	3.73	0.000038	0.245	0.008	0.000237	6.3	partially broken
P3	79.46	10.13	3.76	0.000038	0.252	0.008	0.000244	6.4	partially broken
P4	79.66	10.06	3.75	0.000038	0.242	0.008	0.000234	6.2	partially broken
P5	79.38	10.03	3.80	0.000038	0.247	0.008	0.000239	6.3	partially broken
P6	78.64	8.13	3.79	0.000031	0.200	0.007	0.000193	6.3	
P7	78.57	8.00	3.80	0.000030	0.203	0.007	0.000196	6.4	partially broken
P8	78.63	8.08	3.78	0.000031	0.159	0.007	0.000152	5.0	
P9	78.61	8.15	3.83	0.000031	0.193	0.007	0.000186	6.0	partially broken
P10	78.59	8.13	3.78	0.000031	0.215	0.007	0.000208	6.8	

**Table A19:** Results of the notched impact tests and unnotched impact tests of PO_A,W.

Sample Number	l (mm)	b_B_ (mm)	d (mm)	A (m^2^)	W (J)	W_empty_ (J)	W_corr_ (kJ)	a_cN_ (kJ/m^2^)	Note
P1	78.52	10.11	3.68	0.000037	0.316	0.004	0.000312	8.4	
P2	78.98	10.11	3.82	0.000039	0.388	0.004	0.000384	9.9	partially broken
P3	78.64	10.10	3.70	0.000037	0.308	0.004	0.000304	8.1	partially broken
P4	78.66	10.20	3.68	0.000038	0.396	0.004	0.000392	10.4	partially broken
P5	78.70	10.15	3.65	0.000037	0.396	0.004	0.000392	10.6	partially broken
P6	78.85	8.01	3.67	0.000029	0.234	0.007	0.000227	7.7	
P7	78.86	8.30	3.61	0.000030	0.219	0.007	0.000212	7.1	
P8	78.85	8.13	3.62	0.000029	0.191	0.007	0.000184	6.3	
P9	78.75	8.15	3.65	0.000030	0.217	0.007	0.000210	7.1	
P10	78.70	8.09	3.64	0.000029	0.200	0.007	0.000193	6.6	

**Table A20:** Results of the notched impact tests and unnotched impact tests of PO_A,C.

Sample Number	l (mm)	b_B_ (mm)	d (mm)	A (m^2^)	W (J)	W_empty_ (J)	W_corr_ (kJ)	a_cN_ (kJ/m^2^)	Note
P1	79.55	10.05	3.87	0.000039	0.234	0.008	0.000226	5.8	
P2	78.61	10.03	3.61	0.000036	0.289	0.008	0.000281	7.8	
P3	78.66	9.96	3.75	0.000037	0.311	0.008	0.000303	8.1	
P4	78.37	9.95	3.63	0.000036	0.318	0.008	0.000310	8.6	
P5	78.59	9.81	3.65	0.000036	0.325	0.008	0.000317	8.9	
P6	78.56	8.35	3.65	0.000030	0.073	0.007	0.000066	2.2	
P7	78.51	8.21	3.65	0.000030	0.097	0.007	0.000090	3.0	
P8	78.75	8.62	3.59	0.000031	0.084	0.007	0.000077	2.5	
P9	78.21	8.35	3.68	0.000031	0.086	0.007	0.000079	2.6	
P10	78.60	8.18	3.63	0.000030	0.088	0.007	0.000081	2.7	

**Table A21:** Results of the notched impact tests and unnotched impact tests of PO_A,W,C.

Sample Number	l (mm)	b_B_ (mm)	d (mm)	A (m^2^)	W (J)	W_empty_ (J)	W_corr_ (kJ)	a_cN_ (kJ/m^2^)	Note
P1	78.96	9.90	3.61	0.000036	0.232	0.007	0.000225	6.3	
P2	79.11	9.89	3.85	0.000038	0.245	0.007	0.000238	6.3	
P3	78.71	9.97	3.59	0.000036	0.231	0.007	0.000224	6.3	
P4	78.62	9.98	3.61	0.000036	0.214	0.007	0.000207	5.7	
P5	78.48	10.04	3.57	0.000036	0.321	0.007	0.000314	8.8	
P6	79.21	7.94	3.54	0.000028	0.105	0.007	0.000098	3.5	
P7	79.15	8.14	3.53	0.000029	0.106	0.007	0.000099	3.4	
P8	79.42	8.02	3.88	0.000031	0.106	0.007	0.000099	3.2	
P9	79.10	8.00	3.57	0.000029	0.105	0.007	0.000098	3.4	
P10	79.20	8.08	3.57	0.000029	0.105	0.007	0.000098	3.4	

**Table A22:** Results of the notched impact tests and unnotched impact tests of PO_B.

Sample Number	l (mm)	b_B_ (mm)	d (mm)	A (m^2^)	W (J)	W_empty_ (J)	W_corr_ (kJ)	a_cN_ (kJ/m^2^)	Note
P1	78.79	9.96	3.61	0.000036	0.202	0.008	0.000194	5.4	partially broken
P2	78.55	9.96	3.57	0.000036	0.342	0.008	0.000334	9.4	partially broken
P3	78.90	9.92	3.56	0.000035	0.284	0.008	0.000276	7.8	partially broken
P4	78.42	9.97	3.61	0.000036	0.390	0.008	0.000382	10.6	
P5	78.49	10.02	3.53	0.000035	0.245	0.008	0.000237	6.7	partially broken
P6	78.46	8.08	3.69	0.000030	0.206	0.007	0.000199	6.7	
P7	78.41	7.95	3.50	0.000028	0.183	0.007	0.000176	6.3	partially broken
P8	78.55	7.92	3.59	0.000028	0.147	0.007	0.000140	4.9	partially broken
P9	78.53	7.94	3.52	0.000028	0.195	0.007	0.000188	6.7	
P10	78.39	7.85	3.77	0.000030	0.245	0.007	0.000238	8.0	

**Table A23:** Results of the notched impact tests and unnotched impact tests of PO_B,C.

Sample Number	l (mm)	b_B_ (mm)	d (mm)	A (m^2^)	W (J)	W_empty_ (J)	W_corr_ (kJ)	a_cN_ (kJ/m^2^)	Note
P1	78.64	9.99	3.51	0.000035	0.708	0.004	0.000704	20.1	
P2	78.58	9.93	3.52	0.000035	0.820	0.004	0.000816	23.3	
P3	78.72	10.00	3.56	0.000036	0.676	0.004	0.000672	18.9	
P4	78.72	9.95	3.60	0.000036	0.680	0.004	0.000676	18.9	
P5	78.61	9.99	3.54	0.000035	0.596	0.004	0.000592	16.7	
P6	78.68	8.08	3.56	0.000029	0.121	0.007	0.000114	4.0	
P7	78.70	7.95	3.58	0.000028	0.111	0.007	0.000104	3.7	
P8	78.72	7.92	3.57	0.000028	0.106	0.007	0.000099	3.5	
P9	78.70	7.94	3.55	0.000028	0.113	0.007	0.000106	3.8	
P10	78.81	7.85	3.56	0.000028	0.112	0.007	0.000105	3.8	

**Table A24:** Results of the notched impact tests and unnotched impact tests of P.

Sample Number	l (mm)	b_B_ (mm)	d (mm)	A (m^2^)	W (J)	W_leer_ (J)	W_empty_ (kJ)	a_cN_ (kJ/m^2^)	Note
P1	78.72	10.07	4.08	0.000041	0.162	0.008	0.000154	3.7	parcially broken
P2	79.23	10.16	3.87	0.000039	0.214	0.008	0.000206	5.2	parcially broken
P3	79.14	10.32	3.88	0.000040	0.189	0.008	0.000181	4.5	parcially broken
P4	79.20	10.15	3.87	0.000039	0.151	0.008	0.000143	3.6	parcially broken
P5	79.10	10.17	4.15	0.000042	0.231	0.008	0.000223	5.3	parcially broken
P6	79.00	8.43	3.88	0.000033	0.129	0.007	0.000122	3.7	
P7	79.17	8.05	3.95	0.000032	0.155	0.007	0.000148	4.7	
P8	79.28	8.01	3.93	0.000031	0.135	0.007	0.000128	4.1	
P9	79.24	8.02	3.89	0.000031	0.143	0.007	0.000136	4.4	
P10	79.20	8.01	3.97	0.000032	0.138	0.007	0.000131	4.1	

**Table A25:** Results of the notched impact tests and unnotched impact tests of P_C.

Sample Number	l (mm)	b_B_ (mm)	d (mm)	A (m^2^)	W (J)	W_empty_ (J)	W_corr_ (kJ)	a_cN_ (kJ/m^2^)	Note
P1	79.23	9.92	3.65	0.000036	0.184	0.008	0.000176	4.9	
P2	78.38	10.05	3.72	0.000037	0.132	0.008	0.000124	3.3	
P3	78.42	10.01	3.69	0.000037	0.235	0.008	0.000227	6.1	
P4	78.42	9.97	3.65	0.000036	0.169	0.008	0.000161	4.4	
P5	78.19	9.95	3.66	0.000036	0.188	0.008	0.000180	4.9	
P6	79.47	7.98	3.70	0.000030	0.077	0.007	0.000070	2.4	
P7	78.38	8.00	3.67	0.000029	0.074	0.007	0.000067	2.3	
P8	79.41	7.97	3.69	0.000029	0.078	0.007	0.000071	2.4	
P9	79.55	7.96	3.70	0.000029	0.066	0.007	0.000059	2.0	
P10	79.35	8.06	3.67	0.000030	0.083	0.007	0.000076	2.6	

## Appendix F. Tensile Tests

**Table A26:** Results of the tensile tests of P_PE.

Sample Number	Curve Type	E_t_ (MPa)	s_y_ (MPa)	F_y_ (N)	e_y_ (%)	e_Y_ (mm)	s_m_ (MPa)	s_M_ (N)	e_m_ (%)	e_M_ (mm)	s_b_ (MPa)	s_B_ (N)	e_b_ (%)	e_B_ (mm)	b (mm)	h (mm)	A_0_ (mm^2^)
P1	c	532.0	9.12	325.6	6.06	3.028	9.12	325.6	6.06	3.028	8.227	293.7	7.08	3.54	9.89	3.61	35.70
P2	a	560.6	−	−	−	−	8.67	320.9	4.44	2.219	8.393	310.6	4.93	2.47	10	3.7	37.00
P3	c	579.0	9.19	329.4	5.43	2.714	9.19	329.4	5.43	2.714	1.836	65.9	7.36	3.68	9.88	3.63	35.86
P4	a	550.6	−	−	−	−	8.30	292.5	3.48	1.738	7.969	281.0	3.81	1.90	9.85	3.58	35.26
P5	c	593.3	−	−	−	−	8.61	315.9	3.79	1.897	8.404	308.4	3.93	1.96	9.89	3.71	36.69
P6	c	551.5	9.01	321.2	5.17	2.586	9.01	321.2	5.17	2.586	8.320	296.5	6.13	3.07	9.9	3.6	35.64
**Mean value**		561.2	9.11	325.4	5.55	2.776	8.82	317.6	4.73	2.363	7.192	259.3	5.54	2.77			
**Standard deviation**		21.9	0.09	4.1	0.45	0.227	0.35	13.1	1.00	0.499	2.628	95.4	1.55	0.77			
**Relative deviation [%]**		3.91	0.97	1.27	8.18	8.18	3.93	4.13	21.10	21.10	36.55	36.78	27.94	27.95			

**Table A27:** Results of the tensile tests of P_W,PE.

Sample Number	Curve Type	E_t_ (MPa)	s_m_ (MPa)	F_M_ (N)	e_m_ (%)	e_M_ (mm)	s_b_ (MPa)	s_B_ (N)	e_b_ (%)	e_B_ (mm)	b (mm)	h (mm)	A_0_ (mm^2^)
P1	c	486.5	9.04	321.2	7.21	3.604	6.472	230.0	9.75	4.87	10.04	3.54	35.54
P2	c	498.6	9.13	324.5	5.68	2.842	8.884	315.7	6.00	3.00	10.01	3.55	35.54
P3	c	459.6	9.16	330.1	6.70	3.352	9.163	330.1	6.70	3.35	9.98	3.61	36.03
P4	c	494.7	8.82	314.8	6.63	3.316	8.824	314.8	6.63	3.32	10.05	3.55	35.68
P5	c	483.5	8.12	291.8	3.95	1.973	7.898	284.0	4.16	2.08	10.1	3.56	35.96
P6	c	497.0	8.75	306.7	5.94	2.970	8.753	306.7	5.94	2.97	10.04	3.49	35.04
**Mean value**		486.6	8.84	314.9	6.02	3.010	8.33	296.9	6.53	3.265			
**Standard deviation**		14.5	0.39	13.9	1.16	0.578	1.01	36.1	1.82	0.912			
**Relative deviation [%]**		2.98	4.42	4.41	19.20	19.20	12.08	12.16	27.93	27.93			

**Table A28:** Results of the tensile tests of P_C,PE.

Sample Number	Curve Type	E_t_ (MPa)	s_m_ (MPa)	F_M_ (N)	e_m_ (%)	e_M_ (mm)	s_b_ (MPa)	s_B_ (N)	e_b_ (%)	e_B_ (mm)	b (mm)	h (mm)	A_0_ (mm^2^)
P1	a	540.2	10.89	389.8	9.03	4.513	10.621	380.0	10.30	5.15	9.94	3.6	35.78
P2	a	537.2	11.10	397.3	11.82	5.911	10.593	379.3	15.06	7.53	9.89	3.62	35.80
P3	a	574.2	10.71	388.4	8.67	4.337	10.406	377.4	9.42	4.71	9.91	3.66	36.27
P4	a	557.6	10.58	378.8	6.22	3.111	10.481	375.2	6.41	3.21	9.89	3.62	35.80
P5	a	533.2	10.90	390.8	9.78	4.890	10.517	377.0	11.20	5.60	9.93	3.61	35.85
P6	a	540.8	10.91	391.0	9.70	4.850	10.611	380.3	10.73	5.36	10.01	3.58	35.84
**Mean value**		547.2	10.85	389.4	9.20	4.602	10.538	378.2	10.52	5.26			
**Standard deviation**		15.6	0.18	6.0	1.82	0.912	0.085	2.0	2.80	1.40			
**Relative deviation [%]**		2.86	1.66	1.54	19.82	19.82	0.81	0.52	26.64	26.64			

**Table A29:** Results of the tensile tests of P_W,C,PE.

Sample Number	Curve Type	E_t_ (MPa)	s_y_ (MPa)	F_y_ (N)	e_y_ (%)	e_Y_ (mm)	s_m_ (MPa)	s_M_ (N)	e_m_ (%)	e_M_ (mm)	s_b_ (MPa)	s_B_ (N)	e_b_ (%)	e_B_ (mm)	b (mm)	h (mm)	A_0_ (mm^2^)
P1	c	499.2	12.14	429.7	11.45	5.727	12.14	429.7	11.45	5.727	9.511	336.8	44.26	22.13	9.89	3.58	35.41
P2	c	533.9	12.26	435.7	11.56	5.781	12.26	435.7	11.56	5.781	8.435	299.7	49.63	24.82	9.87	3.6	35.53
P3	c	526.4	12.23	435.4	12.30	6.150	12.23	435.4	12.30	6.150	5.915	210.6	53.86	26.93	9.86	3.61	35.59
P4	c	539.7	12.36	436.6	12.39	6.193	12.36	436.6	12.39	6.193	4.356	153.9	43.94	21.97	9.84	3.59	35.33
P5	c	535.3	12.34	431.0	11.57	5.784	12.34	431.0	11.57	5.784	3.918	136.9	46.26	23.13	9.84	3.55	34.93
P6	c	578.9	12.56	449.8	12.31	6.154	12.56	449.8	12.31	6.154	4.634	166.0	52.04	26.02	9.87	3.63	35.83
**Mean value**		535.6	12.31	436.4	11.93	5.965	12.31	436.4	11.93	5.965	6.128	217.3	48.33	24.17	−	−	−
**Standard deviation**		25.7	0.14	7.2	0.44	0.221	0.14	7.2	0.44	0.221	2.327	82.7	4.15	2.08	−	−	−
**Relative deviation [%]**		4.80	1.16	1.64	3.71	3.71	1.16	1.64	3.71	3.71	37.97	38.07	8.59	8.59	−	−	−

**Table A30:** Results of the tensile tests of P_PP.

Sample Number	Curve Type	E_t_ (MPa)	s_m_ (MPa)	F_M_ (N)	e_m_ (%)	e_M_ (mm)	s_b_ (MPa)	s_B_ (N)	e_b_ (%)	e_B_ (mm)	b (mm)	h (mm)	A_0_ (mm^2^)
P1	a	1373.1	13.09	469.8	1.69	0.843	12.596	452.2	1.84	0.92	10	3.59	35.90
P2	a	1435.4	13.19	473.4	1.64	0.820	13.186	473.4	1.64	0.82	10	3.59	35.90
P3	a	1399.6	11.65	418.2	1.30	0.648	11.648	418.2	1.30	0.65	10	3.59	35.90
P4	a	1414.2	11.88	426.7	1.24	0.620	11.884	426.7	1.24	0.62	10	3.59	35.90
P5	a	1380.5	10.16	364.8	0.97	0.487	10.035	360.3	0.99	0.50	10	3.59	35.90
P6	a	1467.9	11.01	395.4	1.02	0.508	11.014	395.4	1.02	0.51	10	3.59	35.90
**Mean value**		1411.8	11.83	424.7	1.31	0.655	11.727	421.0	1.34	0.67	-	-	-
**Standard deviation**		35.6	1.17	42.2	0.30	0.151	1.121	40.3	0.34	0.17	-	-	-
**Relative deviation [%]**		2.52	9.93	9.93	23.01	23.01	9.56	9.56	25.35	25.35	-	-	-

**Table A31:** Results of the tensile tests of P_W,PP.

Sample Number	Curve Type	E_t_ (MPa)	s_m_ (MPa)	F_M_ (N)	e_m_ (%)	e_M_ (mm)	s_b_ (MPa)	s_B_ (N)	e_b_ (%)	e_B_ (mm)	b (mm)	h (mm)	A_0_ (mm^2^)
P1	a	1509.3	15.69	555.8	1.50	0.748	15.690	555.8	1.50	0.75	10.15	3.49	35.42
P2	a	1517.7	18.16	657.4	2.50	1.250	18.160	657.4	2.50	1.25	10.14	3.57	36.20
P3	a	1508.4	18.65	678.4	2.48	1.242	18.654	678.4	2.48	1.24	10.13	3.59	36.37
P4	a	1552.9	17.90	644.2	2.30	1.150	17.897	644.2	2.30	1.15	10.14	3.55	36.00
P5	a	1452.3	17.58	642.2	2.30	1.150	17.579	642.2	2.30	1.15	10.29	3.55	36.53
P6	a	1502.7	18.21	652.6	2.58	1.292	18.213	652.6	2.58	1.29	10.18	3.52	35.83
**Mean value**		1507.2	17.70	638.4	2.28	1.139	17.699	638.4	2.28	1.14	-	-	-
**Standard deviation**		32.4	1.05	42.5	0.40	0.200	1.047	42.5	0.40	0.20	-	-	-
**Relative deviation [%]**		2.15	5.92	6.66	17.54	17.54	5.92	6.66	17.54	17.54	-	-	-

**Table A32:** Results of the tensile tests of P_C,PP.

Sample Number	Curve Type	E_t_ (MPa)	s_m_ (MPa)	F_M_ (N)	e_m_ (%)	e_M_ (mm)	s_b_ (MPa)	s_B_ (N)	e_b_ (%)	e_B_ (mm)	b (mm)	h (mm)	A_0_ (mm^2^)
P1	a	1697.3	14.46	525.1	1.24	0.620	14.455	525.1	1.24	0.62	9.98	3.64	36.33
P2	a	1573.2	16.34	592.8	1.74	0.872	16.345	592.8	1.74	0.87	9.91	3.66	36.27
P3	a	1599.2	17.61	618.4	1.99	0.995	17.614	618.4	1.99	1.00	9.78	3.59	35.11
P4	a	1481.3	16.23	576.5	1.59	0.794	16.137	573.3	1.59	0.79	9.68	3.67	35.53
P5	a	1589.4	15.35	551.9	1.44	0.720	15.352	551.9	1.44	0.72	9.69	3.71	35.95
P6	a	1549.7	16.31	599.0	1.60	0.798	16.276	597.6	1.60	0.80	9.87	3.72	36.72
**Mean value**		1581.7	16.05	577.3	1.60	0.800	16.030	576.5	1.60	0.80	-	-	-
**Standard deviation**		70.6	1.06	34.0	0.26	0.128	1.060	33.8	0.26	0.13	-	-	-
**Relative deviation [%]**		4.46	6.63	5.89	16.01	16.01	6.61	5.87	16.01	16.01	-	-	-

**Table A33:** Results of the tensile tests of P_W,C,PP.

Sample Number	Curve Type	E_t_ (MPa)	s_y_ (MPa)	F_y_ (N)	e_y_ (%)	e_Y_ (mm)	s_m_ (MPa)	s_M_ (N)	e_m_ (%)	e_M_ (mm)	s_b_ (MPa)	s_B_ (N)	e_b_ (%)	e_B_ (mm)	b (mm)	h (mm)	A_0_ (mm^2^)
P1	a	1484.0	-	-	-	-	23.88	869.9	3.88	1.942	23.880	869.9	3.88	1.94	9.98	3.65	36.43
P2	a	1556.5	-	-	-	-	21.07	765.5	3.28	1.638	21.069	765.5	3.28	1.64	9.9	3.67	36.33
P3	c	1542.1	17.54	638.0	2.14	1.071	17.54	638.0	2.14	1.071	10.456	380.3	2.89	1.45	9.91	3.67	36.37
P4	a	1282.4	-	-	-	-	21.20	778.5	3.57	1.783	21.202	778.5	3.57	1.78	9.87	3.72	36.72
P5	a	2039.3	-	-	-	-	20.64	746.1	3.50	1.749	19.999	722.9	3.72	1.86	9.93	3.64	36.15
P6	a	1616.8	-	-	-	-	20.87	758.9	3.55	1.776	20.867	758.9	3.55	1.78	9.91	3.67	36.37
**Mean value**		1586.9	17.54	638.0	2.14	1.071	20.87	759.5	3.32	1.660	19.579	712.7	3.48	1.74	-	-	-
**Standard deviation**		249.7	-	-	-	-	2.02	74.2	0.61	0.304	4.657	170.0	0.35	0.18	-	-	-
**Relative deviation [%]**		15.74	-	-	-	-	9.66	9.77	18.34	18.34	23.78	23.86	10.09	10.07	-	-	-

**Table A34:** Results of the tensile tests of P_PS.

Sample Number	Curve Type	E_t_ (MPa)	s_y_ (MPa)	F_y_ (N)	e_y_ (%)	e_Y_ (mm)	s_m_ (MPa)	s_M_ (N)	e_m_ (%)	e_M_ (mm)	s_b_ (MPa)	s_B_ (N)	e_b_ (%)	e_B_ (mm)	b (mm)	h (mm)	A_0_ (mm^2^)
P1	a	924.8	-	-	-	-	5.83	216.3	0.78	0.391	5.664	210.1	0.79	0.40	10	3.71	37.10
P2	a	914.5	-	-	-	-	6.01	223.4	0.93	0.464	5.663	210.3	1.01	0.50	10.01	3.71	37.14
P3	c	1005.8	6.24	227.9	0.86	0.429	6.24	227.9	0.86	0.429	1.246	45.5	1.82	0.91	9.95	3.67	36.52
P4	c	909.4	6.25	229.0	1.02	0.510	6.25	229.0	1.02	0.510	1.248	45.8	1.90	0.95	9.91	3.7	36.67
P5	a	983.7	-	-	-	-	6.89	259.1	1.13	0.565	6.647	249.9	1.19	0.60	10	3.76	37.60
P6	a	990.5	-	-	-	-	6.12	219.6	0.78	0.389	5.959	213.8	0.80	0.40	9.91	3.62	35.87
**Mean value**		954.8	6.24	228.5	0.94	0.469	6.22	229.2	0.92	0.458	4.404	162.6	1.25	0.63	-	-	-
**Standard deviation**		43.1	0.00	0.8	0.11	0.057	0.36	15.4	0.14	0.070	2.472	91.8	0.50	0.25	-	-	-
**Relative deviation [%]**		4.52	0.06	0.35	12.11	12.11	5.80	6.71	15.19	15.19	56.13	56.48	39.55	39.55	-	-	-

**Table A35:** Results of the tensile tests of P_W,PS.

Sample Number	Curve Type	E_t_ (MPa)	s_m_ (MPa)	F_M_ (N)	e_m_ (%)	e_M_ (mm)	s_b_ (MPa)	s_B_ (N)	e_b_ (%)	e_B_ (mm)	b (mm)	h (mm)	A_0_ (mm^2^)
P1	a	2265.6	12.65	483.4	0.64	0.318	12.545	479.2	0.64	0.32	10	3.82	38.20
P2	a	2336.9	13.34	511.0	0.69	0.346	12.800	490.4	0.72	0.36	10.03	3.82	38.31
P3	a	2309.6	14.40	547.8	0.74	0.370	14.402	547.8	0.74	0.37	10.01	3.8	38.04
P4	a	2244.1	13.65	527.9	0.69	0.343	13.436	519.6	0.68	0.34	10.07	3.84	38.67
P5	a	2267.7	14.25	559.5	0.78	0.391	14.246	559.5	0.78	0.39	10.2	3.85	39.27
P6	a	2379.6	13.81	536.7	0.65	0.323	13.785	535.5	0.65	0.32	10.09	3.85	38.85
**Mean value**		2300.6	13.68	527.7	0.70	0.348	13.536	522.0	0.70	0.35	-	-	-
**Standard deviation**		51.3	0.64	27.3	0.06	0.028	0.755	31.9	0.06	0.03	-	-	-
**Relative deviation [%]**		2.23	4.66	5.18	8.05	8.05	5.58	6.11	8.04	8.04	-	-	-

**Table A36:** Results of the tensile tests of P_C,PS.

Sample Number	Curve Type	E_t_ (MPa)	s_m_ (MPa)	F_M_ (N)	e_m_ (%)	e_M_ (mm)	s_b_ (MPa)	s_B_ (N)	e_b_ (%)	e_B_ (mm)	b (mm)	h (mm)	A_0_ (mm^2^)
P1	a	2453.1	19.15	771.9	0.95	0.474	18.830	759.1	1.03	0.51	10.18	3.96	40.31
P2	a	2438.1	19.44	773.0	0.96	0.480	19.041	757.2	1.08	0.54	10.17	3.91	39.76
P3	a	2374.3	19.21	786.4	0.95	0.474	17.708	725.1	1.19	0.59	10.16	4.03	40.94
P4	a	2351.4	18.78	769.8	0.90	0.451	18.776	769.8	0.90	0.45	10.25	4	41.00
P5	a	2446.2	19.51	782.2	0.93	0.467	19.461	780.2	0.95	0.48	10.15	3.95	40.09
P6	a	2363.5	18.75	755.2	0.86	0.429	18.751	755.2	0.86	0.43	10.17	3.96	40.27
**Mean value**		2404.4	19.14	773.1	0.92	0.462	18.761	757.8	1.00	0.50	-	-	-
**Standard deviation**		46.1	0.32	10.9	0.04	0.019	0.580	18.6	0.12	0.06	-	-	-
**Relative deviation [%]**		1.92	1.67	1.41	4.12	4.12	3.09	2.45	12.23	12.23	-	-	-

**Table A37:** Results of the tensile tests of P_W,C,PS.

Sample Number	Curve Type	E_t_ (MPa)	s_m_ (MPa)	F_M_ (N)	e_m_ (%)	e_M_ (mm)	s_b_ (MPa)	s_B_ (N)	e_b_ (%)	e_B_ (mm)	b (mm)	h (mm)	A_0_ (mm^2^)
P1	a	930.4	6.80	248.6	0.86	0.432	6.729	245.9	0.87	0.43	9.85	3.71	36.54
P2	a	923.3	7.33	266.0	1.04	0.519	6.714	243.8	1.09	0.55	9.84	3.69	36.31
P3	a	936.5	5.46	196.1	0.67	0.334	4.349	156.1	0.64	0.32	9.78	3.67	35.89
P4	a	896.6	6.33	227.0	0.85	0.425	6.333	227.0	0.85	0.42	9.82	3.65	35.84
P5	a	945.8	6.12	222.7	0.76	0.381	6.119	222.7	0.76	0.38	9.89	3.68	36.40
P6	a	904.8	6.38	229.3	0.88	0.438	6.378	229.3	0.88	0.44	9.85	3.65	35.95
**Mean value**		922.9	6.40	231.6	0.84	0.422	6.104	220.8	0.85	0.42	-	-	-
**Standard deviation**		18.9	0.63	23.8	0.12	0.062	0.891	33.0	0.15	0.08	-	-	-
**Relative deviation [%]**		2.05	9.83	10.28	14.71	14.71	14.60	14.96	17.72	17.72	-	-	-

**Table A38:** Results of the tensile tests of PO_A.

Sample Number	Curve Type	E_t_ (MPa)	s_m_ (MPa)	F_M_ (N)	e_m_ (%)	e_M_ (mm)	s_b_ (MPa)	s_B_ (N)	e_b_ (%)	e_B_ (mm)	b (mm)	h (mm)	A_0_ (mm^2^)
P1	a	994.1	8.87	352.3	1.74	0.872	8.847	351.4	1.81	0.91	10.03	3.96	39.72
P2	a	1043.2	9.59	360.6	1.68	0.838	8.960	337.1	1.82	0.91	10.06	3.74	37.62
P3	a	1113.7	9.44	356.1	1.70	0.852	9.283	350.2	1.79	0.89	9.98	3.78	37.72
P4	a	1115.3	9.50	359.0	1.47	0.733	9.368	354.0	1.50	0.75	9.97	3.79	37.79
P5	a	986.5	9.79	371.1	1.80	0.901	9.449	358.3	1.97	0.99	9.98	3.8	37.92
P6	a	1066.1	9.67	361.7	1.78	0.889	9.241	345.5	1.95	0.98	9.97	3.75	37.39
**Mean value**		1053.2	9.48	360.2	1.70	0.848	9.191	349.4	1.81	0.90	-	-	-
**Standard deviation**		56.1	0.32	6.4	0.12	0.061	0.237	7.4	0.17	0.09	-	-	-
**Relative deviation [%]**		5.32	3.39	1.76	7.14	7.14	2.58	2.11	9.43	9.43	-	-	-

**Table A39:** Results of the tensile tests of PO_A,W.

Sample Number	Curve Type	E_t_ (MPa)	s_m_ (MPa)	F_M_ (N)	e_m_ (%)	e_M_ (mm)	s_b_ (MPa)	s_B_ (N)	e_b_ (%)	e_B_ (mm)	b (mm)	h (mm)	A_0_ (mm^2^)
P1	a	845.8	9.29	341.8	2.19	1.096	8.707	320.4			9.92	3.71	36.80
P2	a	896.9	10.07	358.5	2.66	1.331	9.856	351.0	2.83	1.41	9.92	3.59	35.61
P3	a	855.9	9.77	350.7	2.11	1.054	9.581	343.9	2.17	1.09	9.97	3.6	35.89
P4	a	908.6	9.98	358.3	2.54	1.268	9.763	350.4	2.76	1.38	9.97	3.6	35.89
P5	a	905.5	9.55	342.7	1.87	0.934	9.289	333.1	1.99	0.99	9.99	3.59	35.86
P6	a	860.9	8.95	325.2	2.04	1.018	8.746	317.7	2.20	1.10	9.98	3.64	36.33
**Mean value**		878.9	9.60	346.2	2.23	1.117	9.324	336.1	2.39	1.19	-	-	-
**Standard deviation**		27.8	0.43	12.5	0.31	0.153	0.502	14.7	0.38	0.19	-	-	-
**Relative deviation [%]**		3.16	4.44	3.62	13.65	13.65	5.38	4.37	15.86	15.86	-	-	-

**Table A40:** Results of the tensile tests of PO_A,C.

Sample Number	Curve Type	E_t_ (MPa)	s_m_ (MPa)	F_M_ (N)	e_m_ (%)	e_M_ (mm)	s_b_ (MPa)	s_B_ (N)	e_b_ (%)	e_B_ (mm)	b (mm)	h (mm)	A_0_ (mm^2^)
P1	a	1037.3	10.16	350.1	1.96	0.980	10.158	350.1	1.96	0.98	9.82	3.51	34.47
P2	a	920.6	8.65	306.6	1.43	0.713	8.555	303.2	1.42	0.71	9.9	3.58	35.44
P3	a	835.9	9.37	344.9	2.06	1.029	9.366	344.9	2.06	1.03	9.82	3.75	36.83
P4	a	873.2	9.16	322.3	1.56	0.779	9.161	322.3	1.56	0.78	9.8	3.59	35.18
P5	a	825.4	7.34	258.4	1.11	0.556	7.224	254.3	1.12	0.56	9.78	3.6	35.21
P6	a	840.7	8.70	307.7	1.52	0.760	8.541	302.0	1.53	0.77	9.85	3.59	35.36
**Mean value**		888.9	8.90	315.0	1.61	0.803	8.834	312.8	1.61	0.80	-	-	-
**Standard deviation**		80.5	0.94	33.2	0.35	0.175	0.990	35.1	0.35	0.17	-	-	-
**Relative deviation [%]**		9.06	10.55	10.54	21.85	21.85	11.21	11.20	21.68	21.68	-	-	-

**Table A41:** Results of the tensile tests of PO_A,W,C.

Sample Number	Curve Type	E_t_ (MPa)	s_m_ (MPa)	F_M_ (N)	e_m_ (%)	e_M_ (mm)	s_b_ (MPa)	s_B_ (N)	e_b_ (%)	e_B_ (mm)	b (mm)	h (mm)	A_0_ (mm^2^)
P1	a	814.3	9.52	338.5	1.85	0.925	9.351	332.6	1.87	0.93	9.88	3.6	35.57
P2	a	884.0	10.31	367.3	2.43	1.217	10.310	367.3	2.43	1.22	9.84	3.62	35.62
P3	a	876.0	9.93	352.6	2.05	1.027	9.499	337.4	2.12	1.06	9.84	3.61	35.52
P4	a	860.8	10.19	364.3	2.35	1.174	10.037	358.8	2.40	1.20	9.82	3.64	35.74
P5	a	855.1	9.66	343.3	1.97	0.983	9.663	343.3	1.97	0.98	9.95	3.57	35.52
P6	a	840.7	9.58	338.5	2.03	1.015	9.449	333.7	2.08	1.04	9.92	3.56	35.32
**Mean value**		855.1	9.87	350.7	2.11	1.057	9.718	345.5	2.15	1.07	-	-	-
**Standard deviation**		25.2	0.33	12.8	0.23	0.114	0.377	14.3	0.23	0.11	-	-	-
**Relative deviation [%]**		2.95	3.36	3.64	10.77	10.77	3.88	4.15	10.61	10.61	-	-	-

**Table A42:** Results of the tensile tests of PO_B.

Sample Number	Curve Type	E_t_ (MPa)	s_y_ (MPa)	F_y_ (N)	e_y_ (%)	e_Y_ (mm)	s_m_ (MPa)	s_M_ (N)	e_m_ (%)	e_M_ (mm)	s_b_ (MPa)	s_B_ (N)	e_b_ (%)	e_B_ (mm)	b (mm)	h (mm)	A_0_ (mm^2^)
P1	c	830.6	-	-	-	-	9.47	332.6	1.85	0.925	9.368	329.0	1.86	0.93	10.15	3.46	35.12
P2	c	830.7	-	-	-	-	10.35	384.5	2.52	1.260	10.120	375.8	2.67	1.34	10.2	3.64	37.13
P3	c	877.0	-	-	-	-	9.48	333.7	1.81	0.903	9.302	327.6	1.85	0.92	10.18	3.46	35.22
P4	c	837.6	-	-	-	-	9.35	338.8	1.82	0.910	9.174	332.5	1.85	0.93	10.21	3.55	36.25
P5	c	847.8	10.21	369.3	2.53	1.263	10.21	369.3	2.53	1.263	7.597	274.7	3.20	1.60	10.3	3.51	36.15
P6	c	904.5	-	-	-	-	9.47	350.5	1.58	0.788	9.347	345.9	1.62	0.81	10.25	3.61	37.00
**Mean value**		854.7	10.21	369.3	2.53	1.263	9.72	351.6	2.02	1.008	9.151	330.9	2.18	1.09	-	-	-
**Standard deviation**		29.9	-	-	-	-	0.44	21.2	0.40	0.202	0.832	32.9	0.62	0.31	-	-	-
**Relative deviation [%]**		3.50	-	-	-	-	4.53	6.02	20.05	20.05	9.09	9.95	28.47	28.47	-	-	-

**Table A43:** Results of the tensile tests of PO_B,C.

Sample Number	Curve Type	E_t_ (MPa)	s_y_ (MPa)	F_y_ (N)	e_y_ (%)	e_Y_ (mm)	s_m_ (MPa)	s_M_ (N)	e_m_ (%)	e_M_ (mm)	s_b_ (MPa)	s_B_ (N)	e_b_ (%)	e_B_ (mm)	b (mm)	h (mm)	A_0_ (mm^2^)
P1	a	836.5	-	-	-	-	12.51	435.6	3.64	1.818	12.512	435.6	3.64	1.82	9.89	3.52	34.81
P2	a	845.2	-	-	-	-	11.75	415.5	2.79	1.393	11.689	413.3	2.82	1.41	9.96	3.55	35.36
P3	a	845.3	-	-	-	-	12.43	428.7	3.20	1.600	12.178	419.9	3.34	1.67	9.88	3.49	34.48
P4	c	864.0	12.92	447.9	3.66	1.829	12.92	447.9	3.66	1.829	12.453	431.8	4.27	2.13	9.85	3.52	34.67
P5	a	814.7	-	-	-	-	11.07	384.1	2.23	1.113	11.066	384.1	2.23	1.11	9.86	3.52	34.71
P6	a	870.5	-	-	-	-	12.35	432.6	3.27	1.636	12.043	421.7	3.35	1.68	9.92	3.53	35.02
**Mean value**		846.0	12.92	447.9	3.66	1.829	12.17	424.0	3.13	1.565	11.990	417.7	3.27	1.64	-	-	-
**Standard deviation**		20.0	-	-	-	-	0.66	22.2	0.55	0.273	0.542	18.4	0.70	0.35	-	-	-
**Relative deviation [%]**		2.36	-	-	-	-	5.42	5.24	17.48	17.48	4.52	4.40	21.31	21.31	-	-	-

**Table A44:** Results of the tensile tests of P.

Sample Number	Curve Type	E_t_ (MPa)	s_y_ (MPa)	F_y_ (N)	e_y_ (%)	e_Y_ (mm)	s_m_ (MPa)	s_M_ (N)	e_m_ (%)	e_M_ (mm)	s_b_ (MPa)	s_B_ (N)	e_b_ (%)	e_B_ (mm)	b (mm)	h (mm)	A_0_ (mm^2^)
P1	a	924.8	-	-	-	-	5.83	216.3	0.78	0.391	5.664	210.1	0.79	0.40	10	3.71	37.10
P2	a	914.5	-	-	-	-	6.01	223.4	0.93	0.464	5.663	210.3	1.01	0.50	10.01	3.71	37.14
P3	c	1005.8	6.24	227.9	0.86	0.429	6.24	227.9	0.86	0.429	1.246	45.5	1.82	0.91	9.95	3.67	36.52
P4	c	909.4	6.25	229.0	1.02	0.510	6.25	229.0	1.02	0.510	1.248	45.8	1.90	0.95	9.91	3.7	36.67
P5	c	983.7	-	-	-	-	6.89	259.1	1.13	0.565	6.647	249.9	1.19	0.60	10	3.76	37.60
P6	a	990.5	−	-	-	-	6.12	219.6	0.78	0.389	5.959	213.8	0.80	0.40	9.91	3.62	35.87
**Mean value**		954.8	6.24	228.5	0.94	0.469	6.22	229.2	0.92	0.458	4.404	162.6	1.25	0.63	−	−	−
**Standard deviation**		43.1	0.00	0.8	0.11	0.057	0.36	15.4	0.14	0.070	2.472	91.8	0.50	0.25	−	−	−
**Relative deviation [%]**		4.52	0.06	0.35	12.11	12.11	5.80	6.71	15.19	15.19	56.13	56.48	39.55	39.55	−	−	−

**Table A45:** Results of the tensile tests of P_C.

Sample Number	Curve Type	E_t_ (MPa)	s_m_ (MPa)	F_M_ (N)	e_m_ (%)	e_M_ (mm)	s_b_ (MPa)	s_B_ (N)	e_b_ (%)	e_B_ (mm)	b (mm)	h (mm)	A_0_ (mm^2^)
P1	a	930.4	6.80	248.6	0.86	0.432	6.803	248.6	0.86	0.43	9.85	3.71	36.54
P2	a	923.3	7.33	266.0	1.04	0.519	7.327	266.0	1.04	0.52	9.84	3.69	36.31
P3	a	936.5	5.46	196.1	0.67	0.334	5.465	196.1	0.67	0.33	9.78	3.67	35.89
P4	a	896.6	6.33	227.0	0.85	0.425	6.333	227.0	0.85	0.42	9.82	3.65	35.84
P5	a	945.8	6.12	222.7	0.76	0.381	6.119	222.7	0.76	0.38	9.89	3.68	36.40
P6	a	904.8	6.38	229.3	0.88	0.438	6.378	229.3	0.88	0.44	9.85	3.65	35.95
**Mean value**		922.9	6.40	231.6	0.84	0.422	6.404	231.6	0.84	0.42	−	−	−
**Standard deviation**		18.9	0.63	23.8	0.12	0.062	0.629	23.8	0.12	0.06	−	−	−
**Relative deviation [%]**		2.05	9.83	10.28	14.71	14.71	9.83	10.28	14.71	14.71	−	−	−

## Appendix G. Bulk Densities

**Table A46:** Results of bulk density tests of P_PE; P_C,PE; P_W,PE; and P_W,C,PE.

Material	P_PE		P_C,PE		P_W,PE		P_W,C,PE	
Sample Number	Net Mass (g)	Bulk Density (g/cm^3^)	Net Mass (g)	Bulk Density (g/cm^3^)	Net Mass (g)	Bulk Density (g/cm^3^)	Net Mass (g)	Bulk Density (g/cm^3^)
P1	8.486	0.0849	48.996	0.4900	7.272	0.0727	48.983	0.4898
P2	8.237	0.0824	48.538	0.4854	7.373	0.0737	48.992	0.4899
P3	8.178	0.0818	49.016	0.4902	7.466	0.0747	49.069	0.4907
P4	8.211	0.0821	48.050	0.4805	7.192	0.0719	50.302	0.5030
P5	7.854	0.0785	48.420	0.4842	6.891	0.0689	49.531	0.4953

**Table A47:** Results of bulk density tests of P_PP; P_C,PP; P_W,PP; and P_W,C,PP.

Material	P_PP		P_C,PP		P_W,PP		P_W,C,PP	
Sample Number	Net Mass (g)	Bulk Density (g/cm^3^)	Net Mass (g)	Bulk Density (g/cm^3^)	Net Mass (g)	Bulk Density (g/cm^3^)	Net Mass (g)	Bulk Density (g/cm^3^)
P1	16.825	0.1683	43.877	0.4388	15.463	0.1546	46.074	0.4607
P2	16.368	0.1637	44.926	0.4493	15.625	0.1563	47.104	0.4710
P3	17.537	0.1754	43.697	0.4370	15.075	0.1508	46.296	0.4630
P4	15.492	0.1549	44.057	0.4406	15.196	0.1520	47.222	0.4722
P5	15.415	0.1542	43.769	0.4377	14.955	0.1496	46.712	0.4671

**Table A48:** Results of bulk density tests of P_PS; P_C,PS; P_W,PS; and P_W,C,PS.

Material	P_PS		P_W,PS		P_W,PS		PS_W,C,PS	
Sample Number	Net Mass (g)	Bulk Density (g/cm^3^)	Net Mass (g)	Bulk Density (g/cm^3^)	Net Mass (g)	Bulk Density (g/cm^3^)	Net Mass (g)	Bulk Density (g/cm^3^)
P1	16.986	0.1699	26.825	0.2683	16.369	0.1637	28.51	0.2851
P2	16.08	0.1608	27.184	0.2718	17.167	0.1717	28.062	0.2806
P3	16.506	0.1651	26.986	0.2699	14.616	0.1462	27.355	0.2736
P4	15.464	0.1546	27.859	0.2786	16.297	0.1630	27.326	0.2733
P5	16.216	0.1622	27.357	0.2736	14.762	0.1476	28.576	0.2858

**Table A49:** Results of bulk density tests of PO_A; PO_A,C; PO_A,W; and PO_A,W,C.

Material	PO_A		PO_A,C		PO_A,W		PO_A,W,C	
Sample Number	Net Mass (g)	Bulk Density (g/cm^3^)	Net Mass (g)	Bulk Density (g/cm^3^)	Net Mass (g)	Bulk Density (g/cm^3^)	Net Mass (g)	Bulk Density (g/cm^3^)
P1	6.955	0.0696	40.454	0.4045	7.046	0.0705	46.537	0.4654
P2	7.202	0.0720	39.489	0.3949	6.758	0.0676	45.656	0.4566
P3	7.133	0.0713	40.002	0.4000	6.483	0.0648	45.822	0.4582
P4	6.671	0.0667	39.872	0.3987	6.574	0.0657	45.478	0.4548
P5	7.091	0.0709	40.090	0.4009	6.640	0.0664	44.83	0.4483

**Table A50:** Results of bulk density tests of PO_B; PO_B,C; P; and P_C.

Material	PO_B		PO_B,C		P		P_C	
Sample Number	Net Mass (g)	Bulk Density (g/cm^3^)	Net Mass (g)	Bulk Density (g/cm^3^)	Net Mass (g)	Bulk Density (g/cm^3^)	Net Mass (g)	Bulk Density (g/cm^3^)
P1	8.944	0.0894	47.328	0.4733	10.362	0.1036	44.022	0.4402
P2	8.805	0.0881	48.663	0.4866	11.124	0.1112	44.705	0.4471
P3	9.143	0.0914	48.607	0.4861	11.679	0.1168	44.073	0.4407
P4	9.586	0.0959	47.541	0.4754	12.066	0.1207	44.469	0.4447
P5	9.452	0.0945	47.759	0.4776	11.326	0.1133	44.847	0.4485

**Table A51:** Results of bulk density tests of P_PET and P_W,PET.

Material	P_PET		P_W,PET	
Sample Number	Net Mass (g)	Bulk Density (g/cm^3^)	Net Mass (g)	Bulk Density (g/cm^3^)
P1	24.481	0.2448	18.762	0.1876
P2	24.018	0.2402	16.966	0.1697
P3	23.101	0.2310	19.372	0.1937
P4	24.342	0.2434	18.594	0.1859
P5	24.587	0.2459	17.674	0.1767
**Mean value**		0.2411		0.1827
**Standard deviation**		0.0060		0.0095

## Appendix H. Ash Contents

**Table A52:** Results of ash content tests of P_PET and P_W,PET.

Sample Identification	Crucible Empty (g)	Crucible and Sample (g)	Crucible Containing Ash (g)	Ignition Residue (g)	Ash Content (AC) (%)	Mean Value AC (%)	Relative Deviation (%)
P_PE	36.81	38.90	36.89	0.086	4.08	4.40	0.37
P_PE	38.59	40.46	38.67	0.081	4.32		
P_PE	36.51	38.92	36.62	0.116	4.81		
P_W,PE	34.33	36.30	34.37	0.046	2.31	2.40	0.08
P_W,PE	38.15	40.07	38.19	0.048	2.48		
P_W,PE	33.20	34.84	33.24	0.039	2.40		
P_C,PE	32.97	34.58	33.03	0.063	3.89	3.93	0.14
P_C,PE	38.04	39.99	38.12	0.080	4.09		
P_C,PE	36.40	38.01	36.46	0.061	3.81		
P_W,C,PE	32.72	34.60	32.77	0.044	2.32	2.40	0.08
P_W,C,PE	39.22	40.96	39.26	0.043	2.47		
P_W,C,PE	34.45	36.01	34.48	0.038	2.43		
P_PP	34.92	37.02	34.96	0.045	2.16	2.41	0.23
P_PP	34.89	36.85	34.94	0.051	2.60		
P_PP	35.70	38.24	35.76	0.063	2.48		
P_W,PP	32.67	34.59	32.72	0.045	2.37	2.39	0.59
P_W,PP	35.33	37.33	35.39	0.060	3.00		
P_W,PP	37.50	39.66	37.53	0.040	1.82		
P_C,PP	34.98	37.00	35.04	0.059	2.91	2.67	0.23
P_C,PP	39.22	41.00	39.26	0.044	2.46		
P_C,PP	31.23	32.67	31.27	0.038	2.63		
P_W,C,PP	34.86	36.70	34.89	0.032	1.72	1.68	0.10
P_W,C,PP	34.98	36.63	35.01	0.026	1.57		
P_W,C,PP	35.64	37.28	35.67	0.029	1.77		
P_PS	39.17	41.17	39.25	0.083	4.17	4.59	0.50
P_PS	32.72	34.65	32.81	0.086	4.46		
P_PS	39.48	40.96	39.55	0.076	5.14		
P_W,PS	33.10	35.11	33.17	0.071	3.53	3.36	0.60
P_W,PS	39.98	40.94	40.01	0.026	2.69		
P_W,PS	37.68	39.95	37.77	0.088	3.85		
P_C,PS	39.48	41.24	39.56	0.088	4.97	4.98	0.05
P_C,PS	39.04	41.02	39.14	0.100	5.04		
P_C,PS	31.23	33.28	31.33	0.101	4.94		
P_W,C,PS	38.81	40.94	38.88	0.078	3.63	3.63	0.11
P_W,C,PS	39.81	41.94	39.88	0.075	3.53		
P_W,C,PS	33.64	35.25	33.70	0.060	3.74		
PO_A	34.55	36.60	34.67	0.127	6.18	6.25	0.07
PO_A	33.56	35.43	33.68	0.118	6.32		
PO_A	39.06	41.03	39.18	0.124	6.27		
PO_A,W	36.39	38.37	36.47	0.082	4.13	4.15	0.01
PO_A,W	34.48	36.53	34.56	0.085	4.16		
PO_A,W	32.13	34.51	32.23	0.099	4.15		
PO_A,C	33.17	35.05	33.28	0.110	5.90	5.81	0.08
PO_A,C	34.10	35.73	34.19	0.094	5.76		
PO_A,C	39.17	40.54	39.25	0.079	5.78		
PO_A,W,C	37.67	39.67	37.76	0.082	4.12	3.92	0.21
PO_A,W,C	40.03	41.28	40.08	0.050	3.96		
PO_A,W,C	33.37	34.51	33.42	0.042	3.70		
PO_B	38.58	40.23	38.62	0.036	2.20	2.21	0.10
PO_B	33.44	35.33	33.48	0.040	2.12		
PO_B	37.67	39.87	37.72	0.051	2.31		
PO_B,C	39.04	40.77	39.08	0.043	2.46	2.45	0.05
PO_B,C	34.44	36.07	34.48	0.040	2.48		
PO_B,C	35.64	36.95	35.67	0.031	2.40		
P	33.41	35.43	33.59	0.176	8.71	8.41	0.41
P	34.53	36.85	34.72	0.184	7.95		
P	33.53	36.45	33.78	0.251	8.57		
P_C	36.54	38.42	36.67	0.122	6.47	6.19	0.51
P_C	38.43	40.20	38.54	0.115	6.48		
P_C	38.59	39.76	38.66	0.065	5.60		

## Appendix J. Application Options

**Table A53:** Possible applications for the materials investigated.

Material	Processability	Potential Products for Application
P_PE	compression moulding	distribution pallets [33], bins, pails, roofing [36] and fencing [35] sheets [37], plates for impact sound and thermal insulation, lawn stones
P_W,PE	compression moulding	distribution pallets [33], bins, pails, roofing [36] and fencing [35] sheets [37], plates for impact sound and thermal insulation, lawn stones
P_C,PE	extrusion	Round, square and flat profiles [38], sheets, plates for in-and outdoor applications
P_W,C,PE	extrusion	Round, square and flat profiles [38], sheets, plates for in-and outdoor applications
P_PP	compression moulding	distribution pallets [33], bins, pails, roofing [36] and fencing [35] sheets [37], plates for impact sound and thermal insulation, lawn stones
P_W,PP	compression moulding	distribution pallets [33], bins, pails, roofing [36] and fencing [35] sheets [37], plates for impact sound and thermal insulation, lawn stones
P_C,PP	extrusion	Round, square and flat profiles [38], sheets, plates for in-and outdoor applications
P_W,C,PP	extrusion	Round, square and flat profiles [38], sheets, plates for in-and outdoor applications
P_PET	No processing possible	No application
P_W,PET	No processing possible	No application
P_C,PET	No processing possible	No application
P_W,C,PET	No processing possible	No application
P_PS	compression moulding	roofing and fencing sheets, plates for thermal insulation, office equipment, cases, plant pots, desk items [39]
P_W,PS	compression moulding	roofing and fencing sheets, plates for thermal insulation, office equipment, cases, plant pots, desk items [39]
P_C,PS	extrusion	Round, square and flat profiles [38], sheets, plates for in and outdoor applications
P_W,C,PS	extrusion	Round, square and flat profiles [38], sheets, plates for in and outdoor applications
PO_A	compression moulding	distribution pallets [33], bins, pails, roofing [36] and fencing [35] sheets [37], plates for impact sound and thermal insulation, lawn stones
PO_A,W	compression moulding	distribution pallets [33], bins, pails, roofing [36] and fencing [35] sheets [37], plates for impact sound and thermal insulation, lawn stones
PO_A,C	extrusion	Round, square and flat profiles [38], sheets, plates for in-and outdoor applications
PO_A,W,C	extrusion	Round, square and flat profiles [38], sheets, plates for in- and outdoor applications
PO_B	compression moulding	distribution pallets [33], bins, pails, roofing [36] and fencing [35] sheets [37], plates for impact sound and thermal insulation, lawn stones
PO_B,C	extrusion	Round, square and flat profiles [38], sheets, plates for in-and outdoor applications
P	compression moulding	distribution pallets [33], bins, pails, roofing [36] and fencing [35] sheets [37], plates for impact sound and thermal insulation, lawn stones
P_C	extrusion	Round, square and flat profiles [38], sheets, plates for in-and outdoor applications
